# The impact of hypoxia and glycolysis on liver fibrosis

**DOI:** 10.1186/s12967-026-08147-5

**Published:** 2026-05-16

**Authors:** Yun Li, Fusheng Qin, Hanyang Su, Jianguo Li

**Affiliations:** 1 Department of Gastroenterology, The Fourth Hospital of Changsha, Changsha, Hunan 410006 China; 2 Department of Nephrology, The Fourth Hospital of Changsha, Changsha, Hunan 410006 China; 3 Teaching and Research Section of Clinical Nursing, Xiangya Hospital, Central South University, Changsha, Hunan 410008 China; 4 National Clinical Research Center for Geriatric Disorders, Xiangya Hospital, Central South University, Changsha, Hunan 410008 China

**Keywords:** Liver fibrosis, Lactylation, Glycolysis, Hypoxic response, Fatty acid-binding protein 5, Interferon-stimulated exonuclease gene 20

## Abstract

**Background and methods:**

Hypoxia, glycolysis, and lactylation are key processes in liver fibrosis (LF) and hepatocarcinogenesis, yet the role of hypoxia-, glycolysis-, and lactylation-related genes (HGLRGs) in LF is poorly defined. Here, we systematically analyzed transcriptomic data from LF samples to identify differentially expressed HGLRGs, validate by single-cell transcriptomics, construct co-expression networks, evaluate their biological functions, diagnostic performance, immune relevance, and explore potential therapeutic agents.

**Results:**

Seven HGLRGs—CHST4, FABP5, GPC3, SOX9, SRPX, IFI16, and ISG20—were significantly upregulated in LF and demonstrated strong diagnostic value. FABP5 and ISG20 were consistently elevated in activated stellate cells and human cirrhotic livers. Enrichment analyses indicated that these genes modulate metabolic pathways and drive immune-mediated fibrotic responses. Immune profiling revealed that ISG20 expression inversely correlated with resting NK cell infiltration, and SOX9 with M2 macrophage infiltration. Molecular docking identified seven FDA-approved drugs, including aspirin, Methylene blue, tamoxifen, vorinostat, valproic acid, rosiglitazone and acetaminophen, as potential HGLRG-targeting agents.

**Conclusion:**

HGLRGs are aberrantly upregulated and actively contribute to LF progression through metabolic dysregulation and immune remodeling. FABP5 and ISG20 represents promising biomarkers and therapeutic target. This study providing novel diagnostic markers, drug repurposing opportunities, and strategies for improved clinical management of liver cirrhosis.

**Supplementary Information:**

The online version contains supplementary material available at 10.1186/s12967-026-08147-5.

## Introduction

Liver cirrhosis (LC) remains a major global health burden, contributing substantially to liver-related morbidity and mortality. Between 2012 and 2017, global liver-related deaths exceeded 2.14 million, representing an 11.4% increase since 2012. Meanwhile, the age-standardized incidence rate of cirrhosis reached 25.3 per 100,000 population in 2019 [1, 2]. LC results from irreversible and persistent liver fibrosis (LF), which is primarily driven by chronic liver injury and reparative response. Given its global health impact, there is an urgent need to identify molecular biomarkers, novel therapeutic targets, and drugs that can facilitate early diagnosis, prognostic evaluation, and intervention in LF.

Recent advances have revealed the pivotal roles of hypoxia, glycolysis, and a novel epigenetic modification—lactylation—in regulating immune responses, fibrosis, and tumor progression [3, 4]. In LC, a key pathological event is the activation of hepatic stellate cells (HSCs), which exhibits elevated glycolytic activity and leads to increased lactate production. Lactylation thus bridges metabolic reprogramming and epigenetic regulation, reinforcing HSC activation and fibrosis progression. For instance, lactylation of poly(ADP-ribose) polymerase 1 activates telomere transcription and promotes HBV-driven LF and hepatocellular carcinoma progression10.1016/j.celrep.2025.115457[5]. Moreover, lactylation of histone H3K18 enhances the transcription of pro-fibrotic regulators such as SOX9 [6], while also promoting Th17 cell differentiation and inflammation-associated HSC activation [7]. Targeting key glycolytic enzymes such as hexokinase 2 to inhibit lactate generation has been demonstrated to suppress HSC activation and attenuate liver fibrosis in vivo [8]. These findings suggest lactylation as a critical link between metabolism and epigenetics in LF, highlighting its promise as a molecular target for diagnosis and therapy [9].

However, the transcriptional landscape of hypoxia-, glycolysis-, and lactylation-related genes (HGLRGs) in LF remains poorly characterized, particularly their interplay with the immune microenvironment and their potential as therapeutic targets. Here, we analyzed LF transcriptomic datasets from Gene Expression Omnibus (GEO) to identify differentially expressed HGLRGs, performed functional and immune infiltration analyses, predicted candidate drugs, and validated the expression of FABP5 and ISG20 in activated LX-2 cells and clinical cirrhotic tissues. This integrated approach aims to uncover novel biomarkers and therapeutic targets involved in the metabolic-epigenetic regulation of LF, thereby providing a theoretical basis for early management and anti-fibrotic intervention in LC.

## Methods

### Transcriptomic data processing and identification of HGLRGs in LF

Transcriptomic datasets GSE14323, GSE197112, and GSE84044 were retrieved from the GEO database [10], comprising liver tissue samples from fibrotic and non-fibrotic individuals. Prior to analysis, principal component analysis was used to evaluate batch effects. Where significant, batch correction and normalization were applied using the sva(v3.52.0) and limma(v3.60.6) R packages. Subsequent principal component analysis confirmed the effectiveness of batch correction. Differentially expressed genes (DEGs) were screened using limma, with criteria of |log2FC| > 1.5 and adjusted *p*-value (padj) < 0.05. Heatmaps and volcano plots were generated using pheatmap (v1.0.12) and ggplot2, respectively. A predefined list of 756 HGLRGs, derived from our previous studies [4] was used for further identify LF–associated HGLRGs.

### Identification of LF-associated gene modules via WGCNA

Weighted gene co-expression network analysis (WGCNA) was performed using the WGCNA package (v1.73) to identify gene modules associated with LF. An adjacency matrix was constructed, converted into a topological overlap matrix, and subjected to hierarchical clustering to define modules. Module eigengenes were correlated with clinical phenotype (LF vs. control) to identify relevant modules. Genes from significant modules were intersected with previously identified DEGs and used for subsequent analyses.

### Expression and diagnostic evaluation of HGLRGs

The expression patterns of HGLRGs were validated across the three combined datasets and in an additional non-alcoholic fatty liver disease (NAFLD) cohort (GSE49541) for external validation. Venn diagrams (VennDiagram package, v1.7.3) were used to identify overlapping genes. Violin plots were created using ggpubr (v0.6.0) to visualize differential expression distributions. To evaluate diagnostic performance, receiver operating characteristic (ROC) curves were generated and area under the curve (AUC) values with 95% confidence intervals were calculated for each gene using the pROC package (v1.18.5).

### Single-cell transcriptomics analysis

The single-cell data for LC were sourced from the online Single Cell Portal (https://singlecell.broadinstitute.org/single_cell), utilizing the dataset from patients with alcoholic cirrhosis (SCP2154), which comprises 328,783 cells.

### Co-expression analysis of HGLRGs in LF

Differential expression analysis was conducted using the limma package (v3.60.6), with results visualized via hierarchical clustering heatmaps (pheatmap package, v1.0.12). Subsequently, Pearson correlation coefficients were calculated to assess co-expression patterns, and the resulting correlation matrix was visualized using the corrplot package (v0.95).

### Functional enrichment analysis of HGLRGs

Gene Ontology (GO), Kyoto Encyclopedia of Genes and Genomes (KEGG), and gene set enrichment analysis (GSEA) were performed using the “clusterProfiler” package (v4.12.6) in R. GO and KEGG analyses identified significantly enriched terms and pathways (padj < 0.05) using the Benjamini-Hochberg correction. GSEA was based on RNA-seq data, with genes ranked by correlation to HLGRG expression; pathways with NES > 1 and FDR < 0.25 were considered significant.

### Immune profiling and correlation with HGLRGs

The CIBERSORT algorithm (v1.03) was used to estimate the relative proportions of 22 immune cell types in bulk RNA-seq data, with 1,000 permutations for robust inference. Immune cell proportions were compared between fibrotic and non-fibrotic liver samples, and their correlations with key HGLRGs were assessed using Pearson’s method (*p* < 0.05), visualized via heatmaps. Following this analysis in the combined datasets, an independent LF dataset (GSE49541) was used for validation.

### Molecular docking

Candidate drug-gene interactions were obtained from the DSigDB database [11]. Compound structures and protein structures were retrieved from PubChem (https://pubchem.ncbi.nlm.nih.gov) and UniProt (https://www.uniprot.org), respectively. Molecular docking were performed using the CB-DOCK2 [12, 13]. Binding affinity was assessed by the Vina score, with values < -5 indicating strong binding, -5 to -3 suggesting moderate affinity, and > -3 reflecting weak binding. The lowest-energy conformation was selected to evaluate interaction stability.

### Immunohistochemistry

Human Liver tissue samples were obtained from the Fourth Hospital of Changsha. Non-cirrhotic control tissues were derived from non-neoplastic and non-cirrhotic sources, including hepatic trauma resections, liver sections incidentally collected during cholecystectomy, and hepatic hemangioma resections.

Immunohistochemical staining was conducted according to established protocols [14]. Antigen retrieval was carried out using Tris-EDTA buffer (pH 9.0). The primary antibody against FABP5 (Proteintech, 12348-1-AP) and ISG20 (Abcepta, AP16602c) were used at dilutions of 1:300 and 1:150, respectively. FABP5 and ISG20 expression were evaluated based on staining intensity and abundance. Images were captured using a light microscope at 200× magnification.

### Quantitative real-time PCR assay

The human LX-2 cells were kindly provided by Zhang Xiangqian (Xiangya Hospital) and authenticated by STR profiling. LX-2 cells were activated by TGF-β (10 ng/mL). mRNA extraction was performed as previously described [14]. The primers used were as follows: FABP5 (F: 5′-AGGAGTGGGAATAGCTTTGCG-3′; R: 5′-TGATGCTGAACCAATGCACC-3′) and β-actin (F: 5′-TGCCCATCTACGAGGGGTAT-3′; R: 5′-GGCCATCTCTTGCTCGAAGT-3′).

### Western blotting

LX-2 cells were transfected with either an empty vector or a pCDA3.1-ISG20 plasmid using Lipofectamine 3000 (Thermo Fisher, L3000001). Sample preparation and processing were performed according to previously described protocols [14]. Primary antibodies included rabbit anti-ISG20 (Abcepta, AP16602c), mouse anti-α-SMA (Servicebio, GB12045), and rabbit anti-GAPDH (Goodhere, AB-P-R001), all used at a 1:1000 dilution.

### Statistical analysis

All statistical analyses were performed using R software (version 4.4.1, https://www.r-project.org). DEGs were identified using the limma package (version 3.60.6) with thresholds of log2 fold change > 1.5 and padj < 0.05. A *p* value < 0.05 was considered statistically significant.

## Results

### Data integration and identification of HGLRGs in LF

**Fig. 1:**
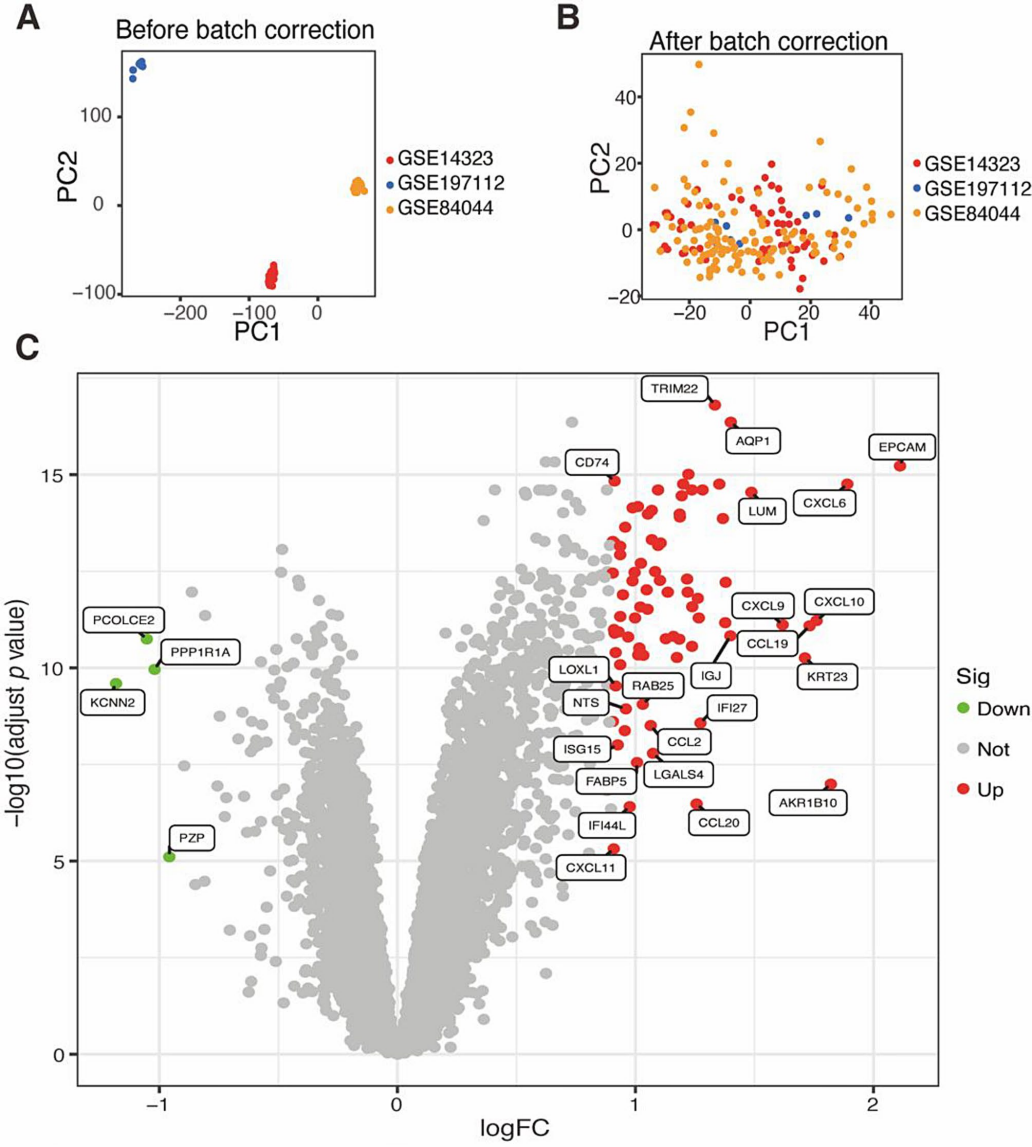
Principal component analysis and differential expression analysis of LF data from GEO. (**A**) Principal component analysis plot before batch correction. Each dot represents a dataset from a different study: GSE14323 (red), GSE197112 (blue), and GSE84044 (orange), which are distinctly separated. (**B**) Principal component analysis plot after batch correction. (**C**) Volcano plot showing differentially expressed HGLRGs. Red dots indicate upregulated genes, while green dots indicate downregulated genes. The gray dots represent genes with no significant change

Three datasets, GSE14323, GSE84044, and GSE197112, were included, comprising 86 non-fibrotic controls and 106 fibrotic liver tissues. Principal component analysis revealed apparent batch effects across datasets (Fig. 1A), which were substantially corrected following normalization using the sva package (Fig. 1B). Significant alterations in hypoxia-glycolysis-lactylation-relaed genes between non-fibrotic and fibrotic liver tissues were visualizedin a volcano plot (Fig. 1C).

**Fig. 2:**
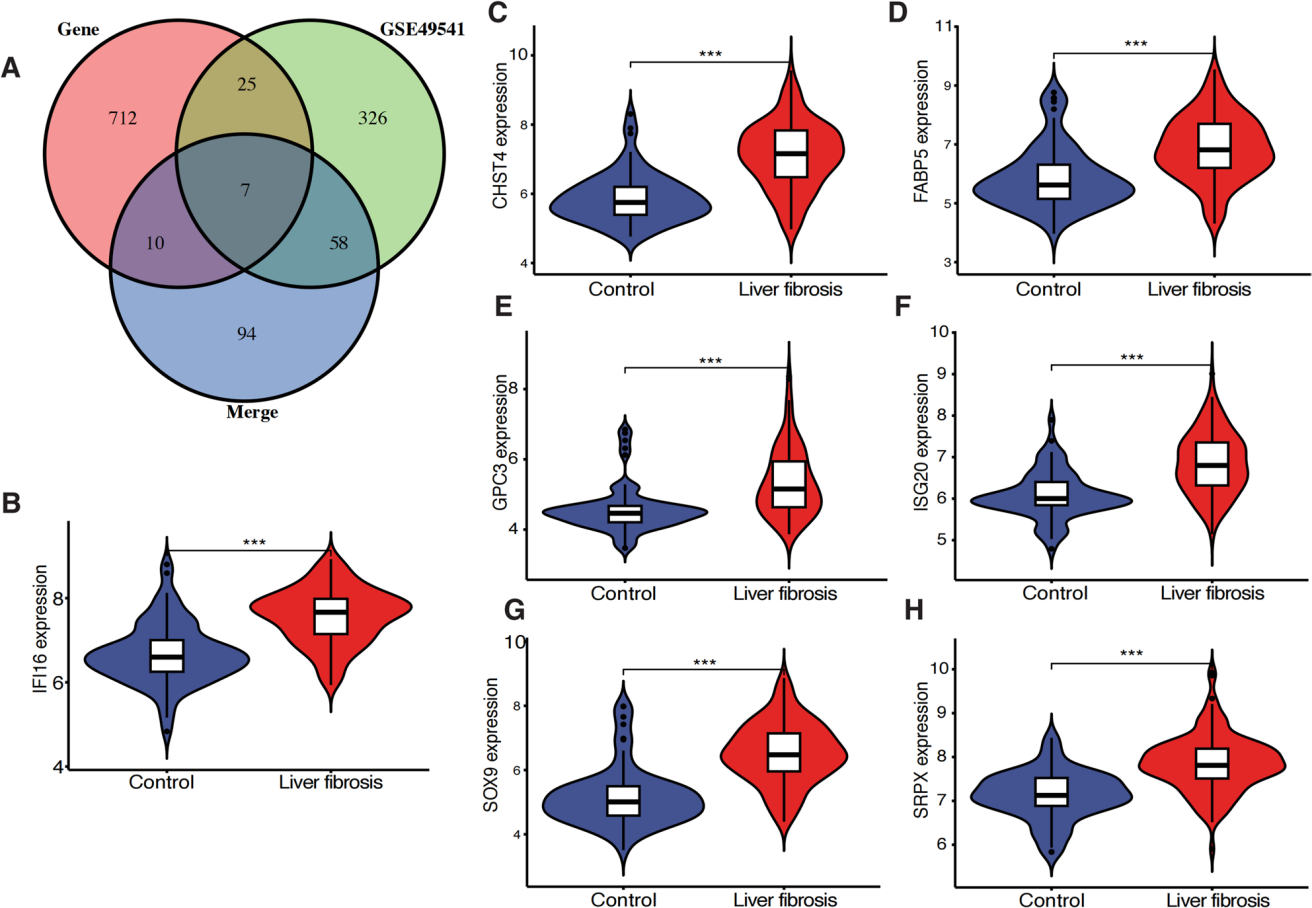
Identification and expression of differentially expressed HGLRGs in LF. (**A**) Venn diagram showing the overlap of differentially expressed genes between the HGLRGs gene list (orange), the genomic dataset GSE49541 (green), and the merge LF group (blue). (**B**-**H**) Violin plots showing the expression levels of selected genes in LF and control groups. Expression of IFI16 (**B**), CHST1 (**C**), FABP5 (**D**), GPC3 (**E**), ISG20 (**F**), SOX9 (**G**), and SRPX (**H**) are significantly higher in HGLRGs compared to controls, with *** indicating a *p* value < 0.001.

WGCNA identified multiple gene modules labeled by color (Supplementary Fig. 1A). Module-trait relationship analysis revealed that the turquoise and brown modules showed the strongest correlations with LF (Supplementary Fig. 1B). Integration of WGCNA results and differentially expressed HGLRGs identified seven consistently upregulated genes in fibrosis: SRY-related HMG-box transcription factor 9 (SOX9), Glypican-3 (GPC3), interferon-stimulated exonuclease gene 20 (ISG20), fatty acid-binding protein 5 (FABP5), Carbohydrate sulfotransferase 4 (CHST4), Sushi repeat-containing protein X-linked (SRPX), and interferon gamma inducible protein 16 (IFI16) (Fig. 2A). Violin plots confirmed their elevated expression in fibrotic tissues (Fig. 2B-H). Supplementary Fig. 2 presents a line plot illustrating consistently upregulated of 7 HGLRGs in LF samples. To validate the generalizability of the seven HGLRGs, a new dataset (GSE49541), comprising a metabolic-associated liver disease cohort, was further analyzed. Expression of FABP5, CHST4, GPC3, IFI16, SOX9, and SRPX remained consistent with previous findings and showed statistically significant differences (Supplementary Fig. 3A-F). For ISG20, a non-significant increasing trend was observed in the fibrosis group, but it did not show a statistically significant difference compared to controls (Supplementary Fig. 3G).

**Fig. 3:**
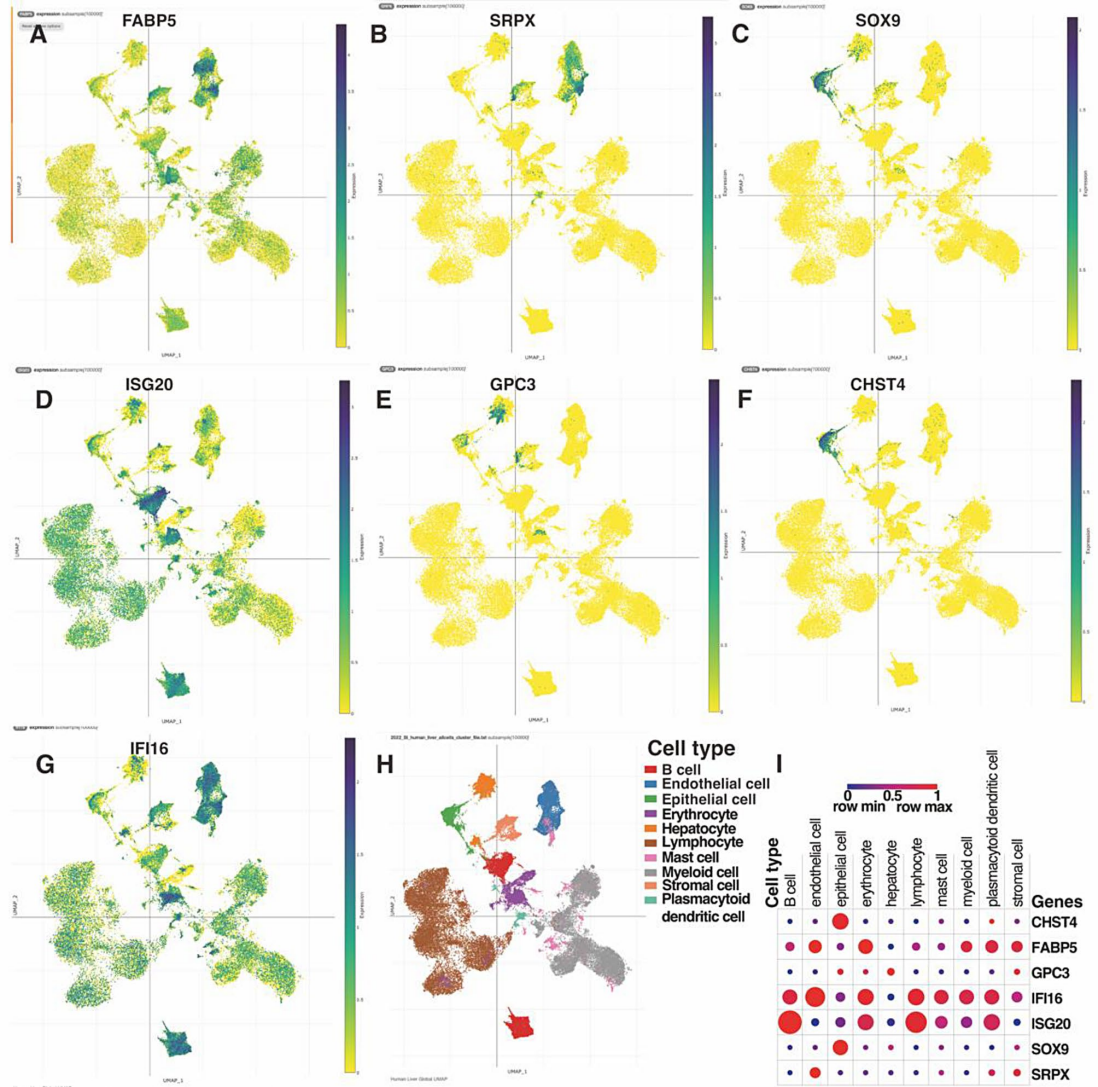
Seven HGLRGs in human liver fibrosis scRNAseq atlas. (**A**-**G**) Distribution of 7 HGLRGs expression across different cell types in liver fibrosis tissues. Each panel represents a different gene: (**A**) FABP5, (**B**) SRPX, (**C**) SOX9, (**D**) ISG20, (**E**) GPC3, (**F**) CHST4, and (**G**) IFI16. The color gradients indicate the expression levels of the genes, with darker colors representing higher expression. (**H**) UMAP visualization of the cellular landscape and annotation of cell types. (**I**) Dot plot showing the expression levels of the 7 HGLRGs across various cell types

Single-cell transcriptomic analysis revealed distinct cellular distributions of these HGLRGs in cirrhotic tissue: FABP5 was primarily in endothelial cells, erythrocytes, stromal cells, B cells, and myeloid cells; SRPX in endothelial cells and stromal cells; SOX9 in endothelial cells and hepatocytes; ISG20 in B cells, lymphocytes, erythrocytes, endothelial cells, plasmacytoid dendritic cells, myeloid cells, and hepatocytes; GPC3 in erythrocytes, epithelial cells, hepatocytes, and stromal cells; CHST4 exclusively in epithelial cells; and IFI16 widely expressed, especially in endothelial, B, erythroid, and lymphocyte populations (Fig. 3A-G). Their expression relationships are shown in Fig. 3I. In summary, these 7 HGLRGs show consistently elevated expression in fibrotic liver tissues, meaning their potential as fibrosis-associated biomarkers.

### Experimental validation of FABP5 and ISG20 expression in LC

**Fig. 4:**
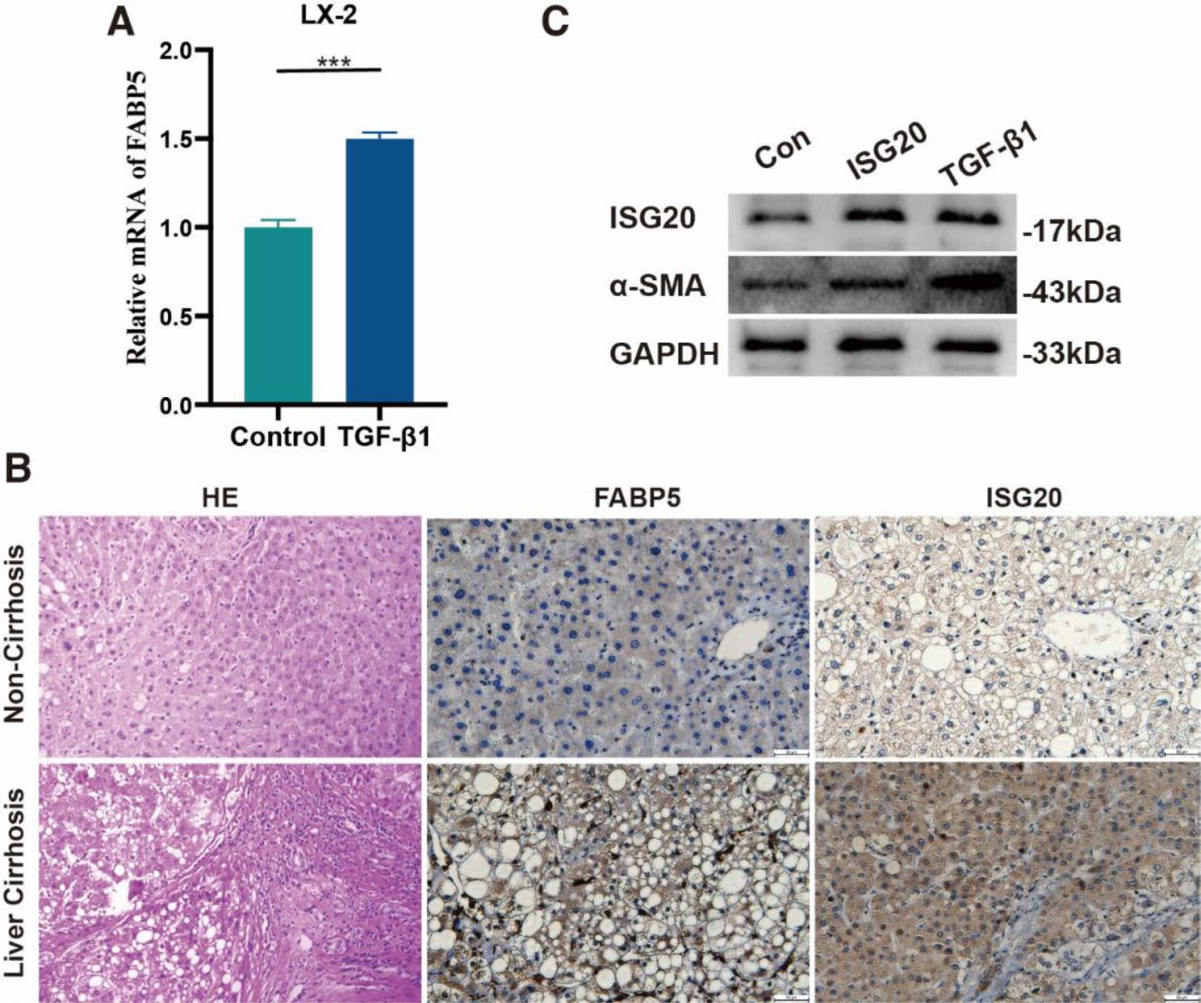
Elevated expression of FABP5 and ISG20 in activated LX-2 cells and cirrhotic liver tissues. (**A**) qPCR analysis showing changes in FABP5 mRNA expression in LX-2 cells after activation by TGF-β stimulation. (**B**) Representative images of HE staining and immunohistochemical staining for FABP5 and ISG20 in liver tissues from cirrhotic patients versus non-cirrhotic controls, observed under 20× magnification. (**C**) Immunoblotting detecting the changes of ISG20 and α-SMA in LX-2 cells after TGF-β stimulation or ISG20 overexpression

To evaluate the clinical relevance of the 7 identified HGLRGs in LC, FABP5 and ISG20 were selected for validation in vitro and in vivo. FABP5 mRNA levels were elevated in activated LX-2 cells (Fig. 4A). In human LC tissues, FABP5 protein expression was strongly positive, compared to weak staining in non-cirrhotic controls (Fig. 4B). Meanwhile, ISG20 expression was also markedly increased in cirrhotic tissues (Fig. 4B). Overexpression of ISG20 in hepatic stellate cells enhanced α-SMA levels, suggesting its role in LX-2 activation (Fig. 4C). Together, these findings support the upregulation of FABP5 and ISG20 in LF and indicate theirs potential involvement in LF pathogenesis.

### Diagnostic evaluation of HGLRGs in LF

**Fig. 5:**
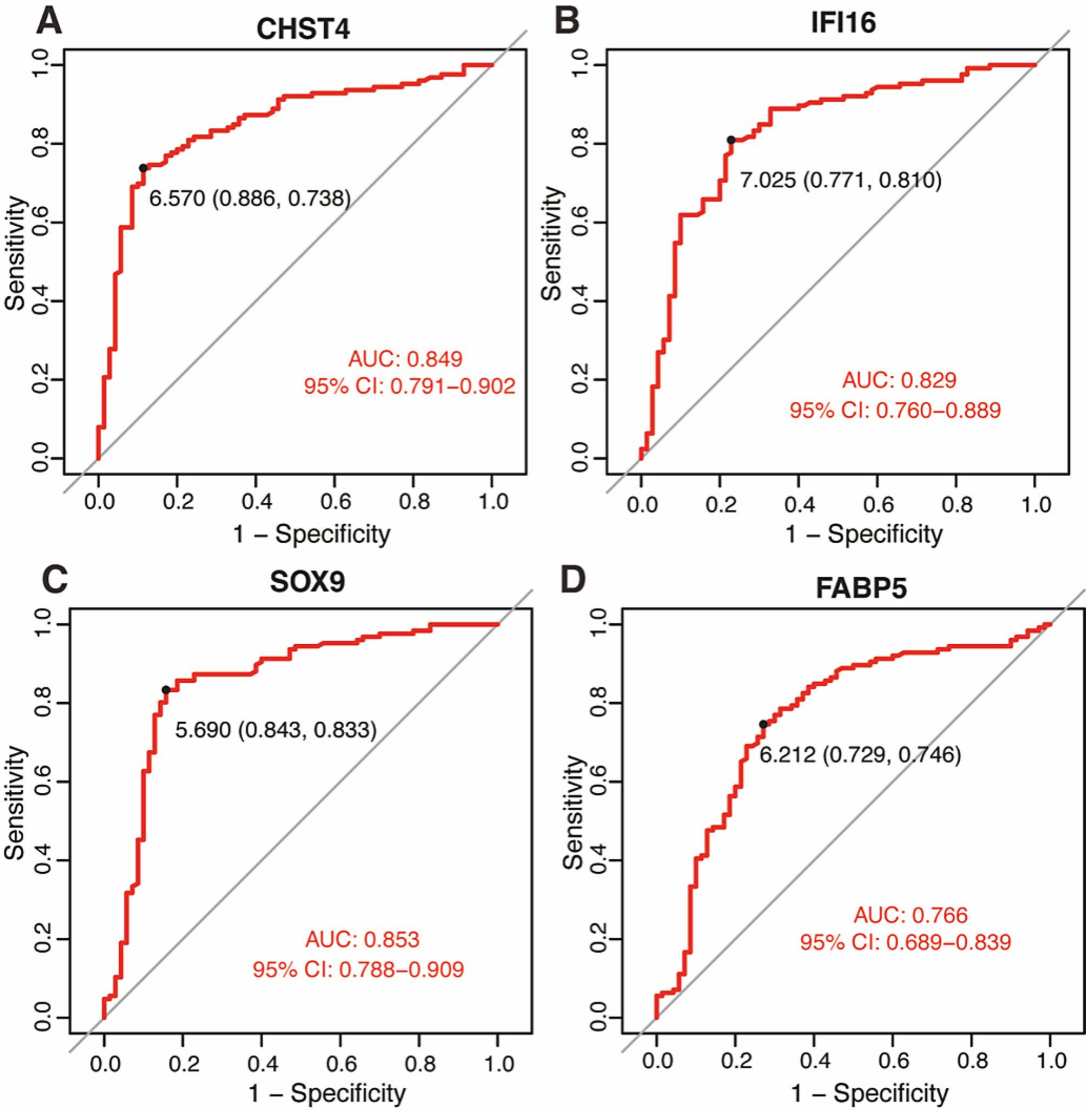
ROC curve analyses of CHST4, IFI16, SOX9 and FABP5 in LF. ROC curves were plotted to evaluate the diagnostic performance of four DEGs in LF patients. The corresponding AUC values were 0.849 for CHST4 (**A**), 0.829 for IFI16 (**B**), 0.853 for SOX9 (**C**) and 0.766 for FABP5 (**D**)

ROC analysis was used to assess the diagnostic performance of screened HGLRGs. Notably, CHST4, IFI16, SOX9, FABP5, ISG20, GPC3, and SRPX showed moderate to high diagnostic accuracy, with AUC values of 0.849, 0.829, 0.853, 0.766, 0.797, 0.768, and 0.819 respectively (Fig. 5 and Supplementary Fig. 4). Furthermore, we evaluated the diagnostic performance of combined HGLRGs. As shown in Supplementary Table 1, several two-gene combinations—such as ISG20 with CHST4 or SOX9, CHST4 with IFI16, SOX9, or SRPX, SRPX with SOX9 or IFI16, and SRPX with IFI16—achieved AUC values more than 0.860. For three-gene combinations, twelve panels, including the combination of ISG20, SRPX, and CHST4, demonstrated high predictive value, all with AUCs greater than 0.863. Collectively, these findings indicate that both individual HGLRGs and their multi-gene combinations may serve as promising diagnostic biomarkers for LF.

### Co-expression patterns and functional relevance of HGLRGs

**Fig. 6:**
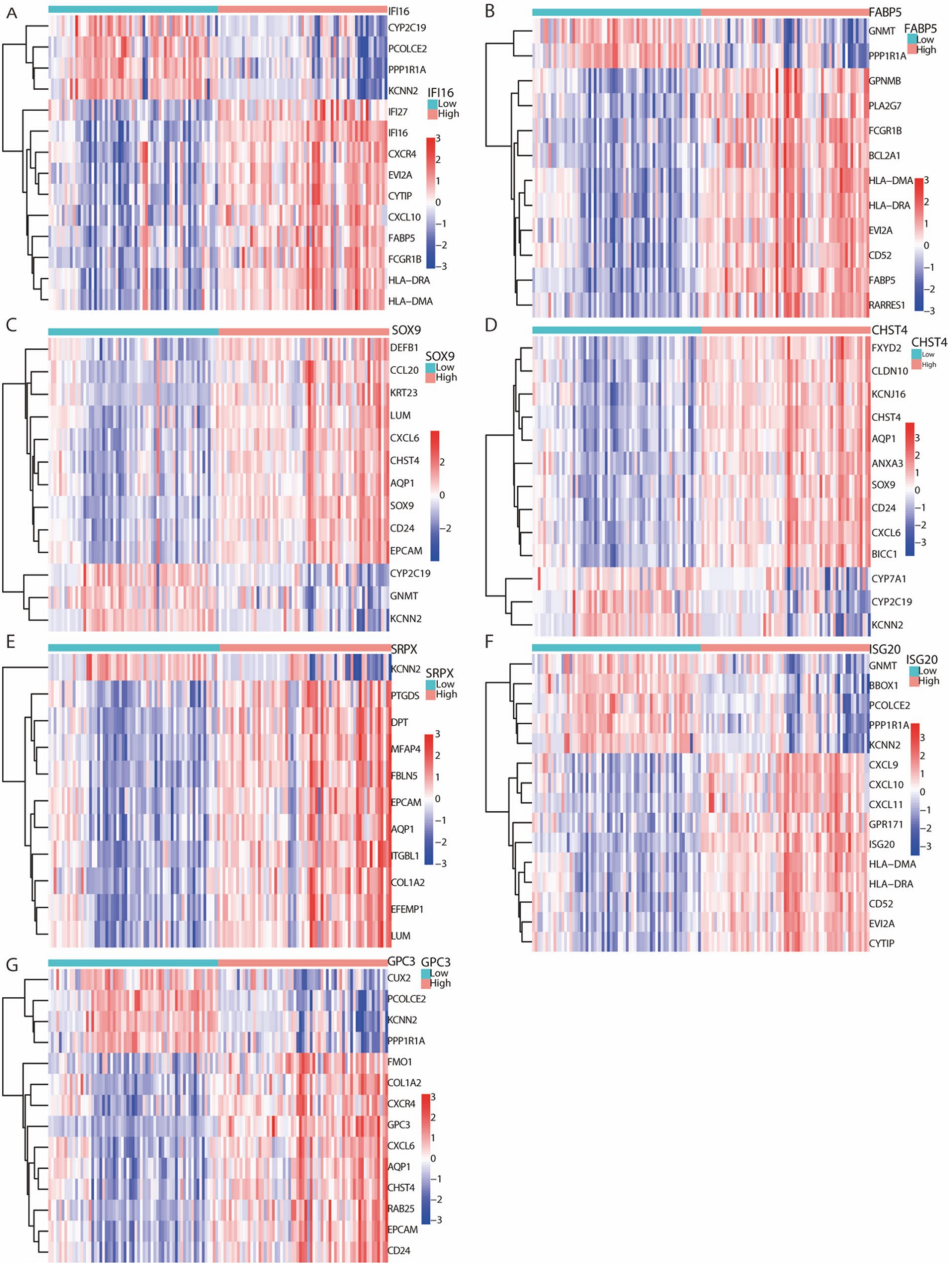
Heatmap of HGLRGs and their co-expressed genes in LF. (**A**) Heatmap illustrates the expression levels of genes closely related to IFI16 in LF and their expression relationship with IFI16. Accordingly, Figures **B**-**G** respectively display the expression levels of associated genes related to FABP5, SOX9, CHST4, SRPX, ISG20, and GPC3

**Fig. 7:**
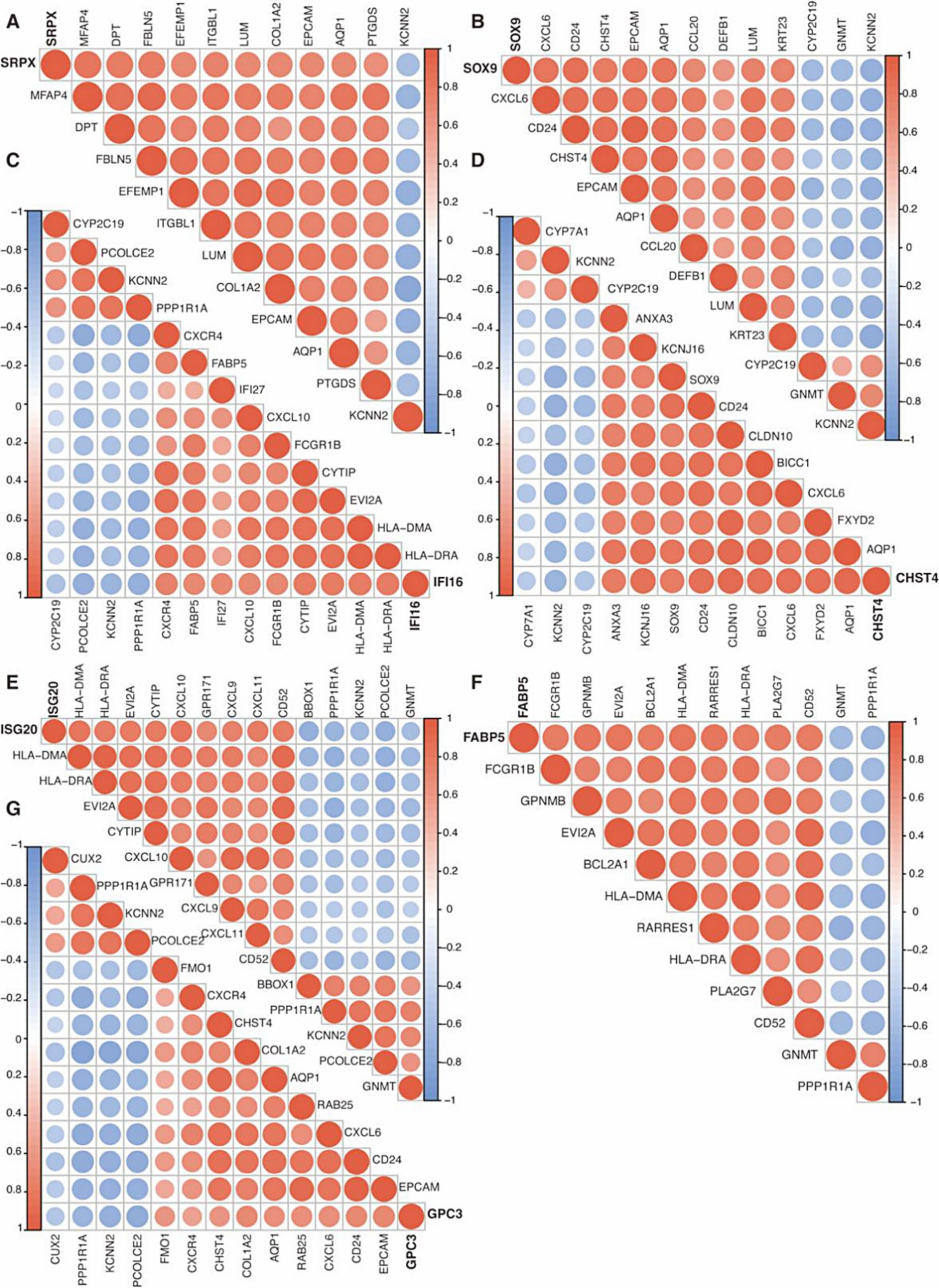
Correlation analysis between HGLRGs and their co-expressed genes. Bubble plots (**A**–**G**) show Spearman correlation coefficients between each of the seven target genes—SRPX (**A**), SOX9 (**B**), IFI16 (**C**), CHST4 (**D**), ISG20 (**E**), FABP5 (**F**), and GPC3 (**G**)—and their closely related genes. The color intensity and size of each circle represent the strength and direction of the correlation (red: positive; blue: negative; larger size indicates stronger correlation). All correlation coefficients were calcuated using normalized gene expression data from fibrotic liver tissues

To explore the transcriptional network of HGLRGs in LF, we conducted a comprehensive co-expression analysis. Heatmaps demonstrated significant co-expression involving IFI16, FABP5, SOX9, CHST4, SRPX, ISG20, and GPC3 in LF tissues (Fig. 6). Positive co-expressions were observed between IFI16 and FABP5, SOX9 and CHST4, as well as CHST4 and GPC3, suggesting cooperative regulation. In contrast, PPP1R1A showed consistent negative correlations with multiple HGLRGs, including IFI16, FABP5, ISG20, and GPC3, implying an inverse regulatory relationship. AQP1 exhibited a strong positive correlation with SOX9, CHST4, SRPX, and GPC3, implicating its involvement in fibrosis-associated pathways. Bubble plot correlation analyses based on Spearman coefficients highlighted the top co-expressed genes associated with each HGLRG (Fig. 7), enhacing their potential functional interplay during LF progression.

### Enrichment of HGLRGs in biological and pathway functions

**Fig. 8:**
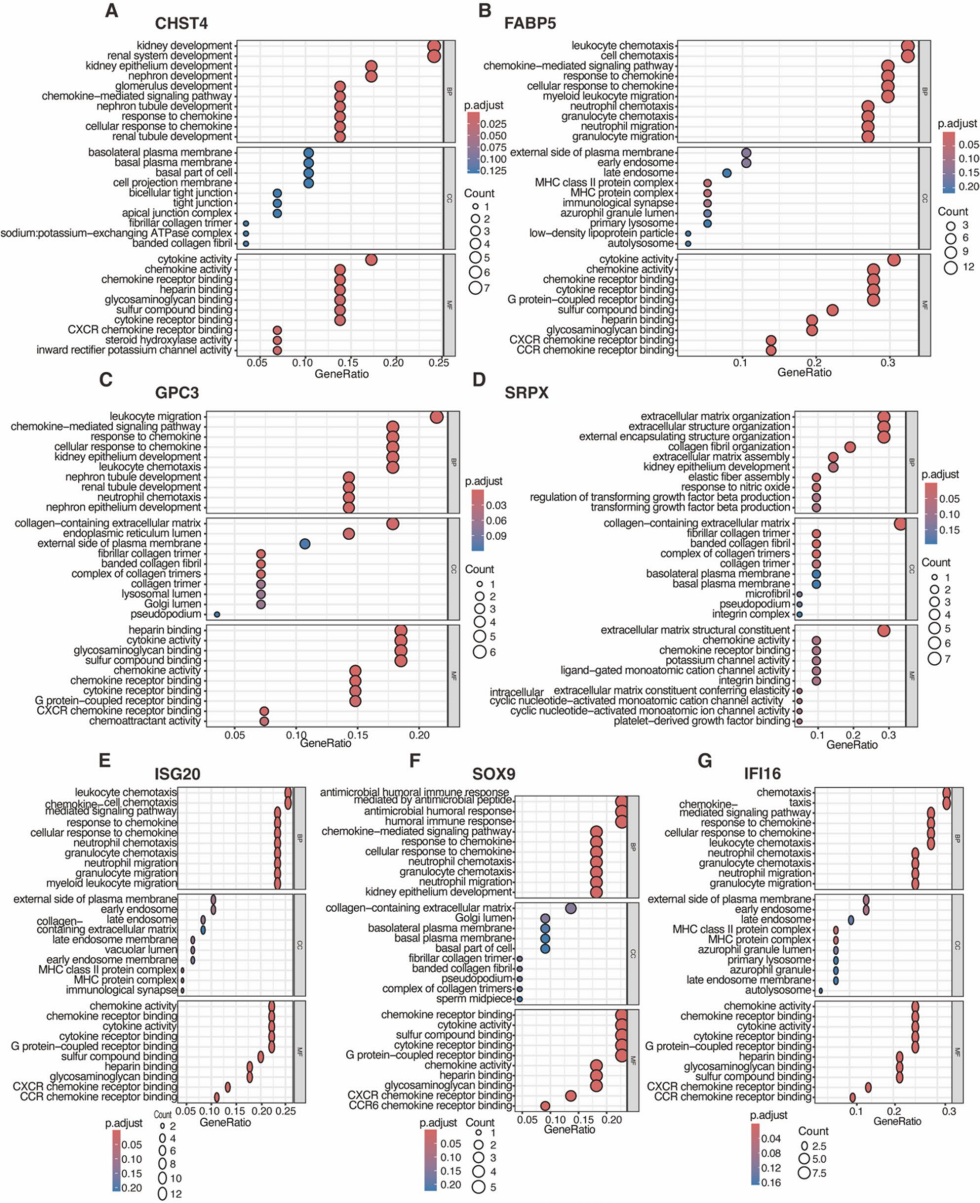
GO enrichment analysis of HGLRGs in LF. GO functional enrichment results for seven key HGLRGs: (**A**) CHST4, (**B**) FABP5, (**C**) GPC3, (**D**) SRPX, (**E**) ISG20, (**F**) SOX9, and (**G**) IFI16. The bubble plots display enriched GO terms across three categories: biological process (BP), cellular component (CC), and molecular function (MF). The x-axis represents the gene ratio, and the y-axis shows the GO terms. The size of each bubble indicates the number of genes involved in each term, while the color gradient reflects the adjusted *p*-value with red indicating higher significance

**Fig. 9:**
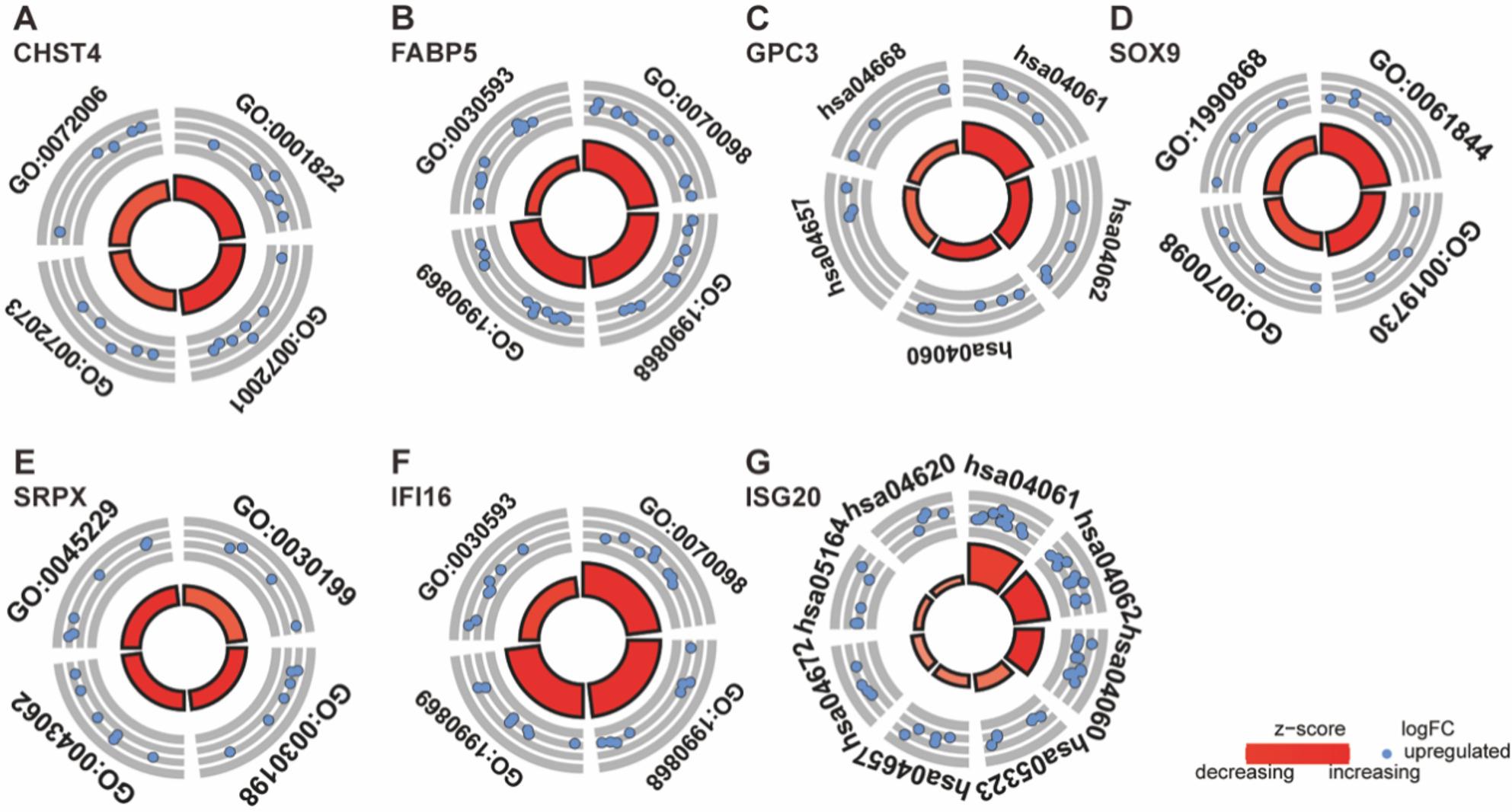
KEGG pathway-based gene-concept network analysis for HGLRGs in LF. Circular gene-concept network plots for the seven key differentially expressed genes: (**A**) CHST4, (**B**) FABP5, (**C**) GPC3, (**D**) SOX9, (**E**) SRPX, (**F**) IFI16, and (**G**) ISG20. Each sector represents an enriched KEGG pathway associated with the respective gene. Red segments in the inner ring indicate the z-score of the pathway, with darker red corresponding to higher enrichment levels. Blue dots in the outer rings represent individual genes contributing to the enrichment, where dot size corresponds to gene log fold change (logFC); all indicated genes are upregulated. Abbreviations: GO:0001822, kidney development; GO:0030198, extracellular matrix organization; GO:0030199, collagen fibril organization; GO:0030593, neutrophil chemotaxis; GO:0043062, extracellular structure organization; GO:0045229, external encapsulating structure organization; GO:0072001, renal system development; GO:0070098, chemokine-mediated signaling pathway; GO:0072006, nephron development; GO:0072073, kidney epithelium development; GO:1,990,868, response to chemokine; GO:1,990,869, cellular response to chemokine; hsa04060 Cytokine–cytokine receptor interaction; hsa04061, Viral protein interaction with cytokine and cytokine receptor; hsa04062, Chemokine signaling pathway; hsa04620, Toll−like receptor signaling pathway; hsa04657, IL-17 signaling pathway; hsa04668, TNF signaling pathway; hsa04672, Intestinal immune network for IgA production; hsa05150, Staphylococcus aureus infection; hsa05164, Influenza A; hsa05169, Epstein−Barr virus infection; hsa05321, Rheumatoid arthritis; hsa05323, Rheumatoid arthritis

To elucidate the biological roles, metabolic processes, and signaling pathways associated with the seven HGLRGs in LF, GO and KEGG enrichment analyses were performed. GO analysis revealed significant enrichment in immune activation and extracellular matrix organization—hallmark features of HSC activation and fibrosis (Fig. 8). Cellular components were predominantly associated with the plasma membrane and lysosome, while molecular functions included cytokine activity and oxidoreductase activity, further highlighting their roles in inflammation and metabolism. KEGG analysis showed involvement of each HGLRG in fibrogenic and immunoregulatory signaling cascades. Specifically, CHST4, GPC3, and IFI16 were enriched in pathways such as chemokine signaling, NOD-like receptor signaling, and ECM–receptor interaction (Fig. 9).

GSEA was conducted to further explore the potential biological roles of the seven HGLRGs in LF. In the high-expression groups of CHST4, FABP5, GPC3, IFI16, SOX9, SRPX, and ISG20, there was consistent enrichment in immune-related and fibrogenic pathways, including cytokine–cytokine receptor interaction, ECM–receptor interaction, focal adhesion, and antigen processing and presentation. Several genes (e.g., FABP5, IFI16, ISG20) were also enriched in autoimmune disease and host-pathogen interaction pathways such as graft-versus-host disease and leishmaniasis, indicating their involvement in immune activation and inflammatory responses during fibrogenesis. In contrast, the low-expression groups of these genes were predominantly enriched in metabolic pathways, including fatty acid metabolism, glycine, serine, and threonine metabolism, peroxisome, and retinol metabolism. Supplementary Fig. 5 presents GSEA results for two representative HLGRGs, CHST4 and FABP5. These results suggest that HGLRGs downregulation may disrupt metabolic homeostasis, while their upregulation may promote immune-mediated fibrotic progression in LF.

### Immune microenvironment landscape of HGLRGs

**Fig. 10:**
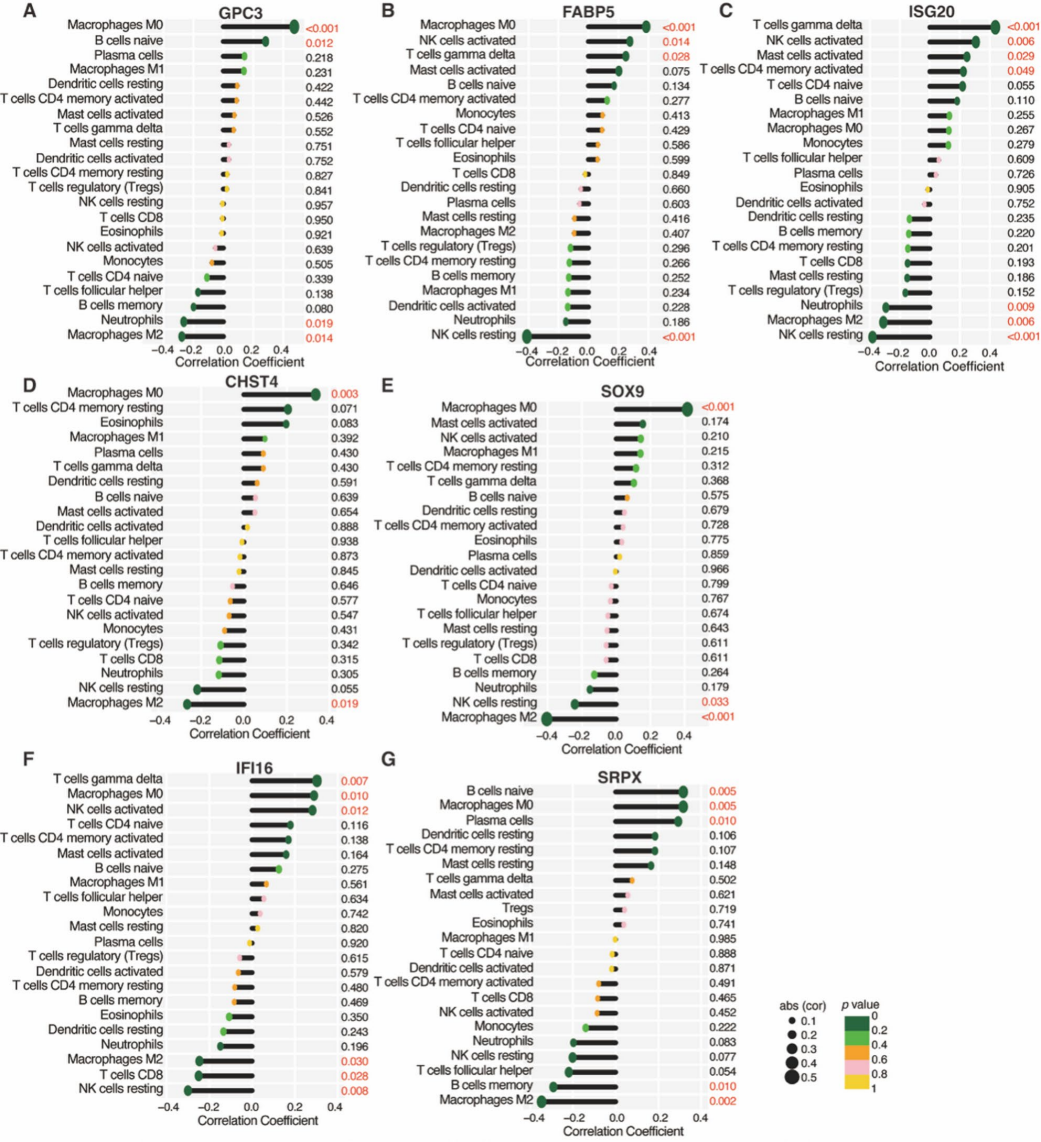
Correlation between hub HGLRGs expression and immune cell infiltration in combined datasets of LF. Spearman correlation coefficients were calculated between the expression levels of the seven hub genes (**A**) GPC3, (**B**) FABP5, (**C**) ISG20, (**D**) CHST4, (**E**) SOX9, (**F**) IFI16, and (**G**) SRPX, and the estimated infiltration levels of 22 immune cell types using CIBERSORT. The dot color indicates the absolute value of the correlation coefficient, and the size of the dot reflects the statistical significance. *p* < 0.05 are labeled in red

Given the strong association between HGLRGs and immune-related pathways identified in enrichment analyses, the immune landscape of LF was further investigated using CIBERSORT. The proportions of 22 immune cell types were estimated in fibrotic and non-fibrotic liver samples. A correlation heatmap revealed complex interactions—both positive and negative—among immune cell populations (Supplementary Fig. 6). Immune infiltration analysis revealed distinct correlations between HGLRG expression levels and the abundance of 22 immune cell types (Fig. 10 and Supplementary Fig. 7), suggesting their role in shaping the hepatic immune microenvironment during fibrosis. Validation in an independent dataset (GSE49541) confirmed a negative correlation between ISG20 expression and resting NK cells infiltration, as well as between SOX9 and M2 macrophage infiltration (Supplementary Fig. 8), supporting a role for SOX9 and ISG20 in modulating LF through immune microenvironment regulation.

### Surface interaction landscapes screening candidate drugs targeting HGLRGs

**Fig. 11:**
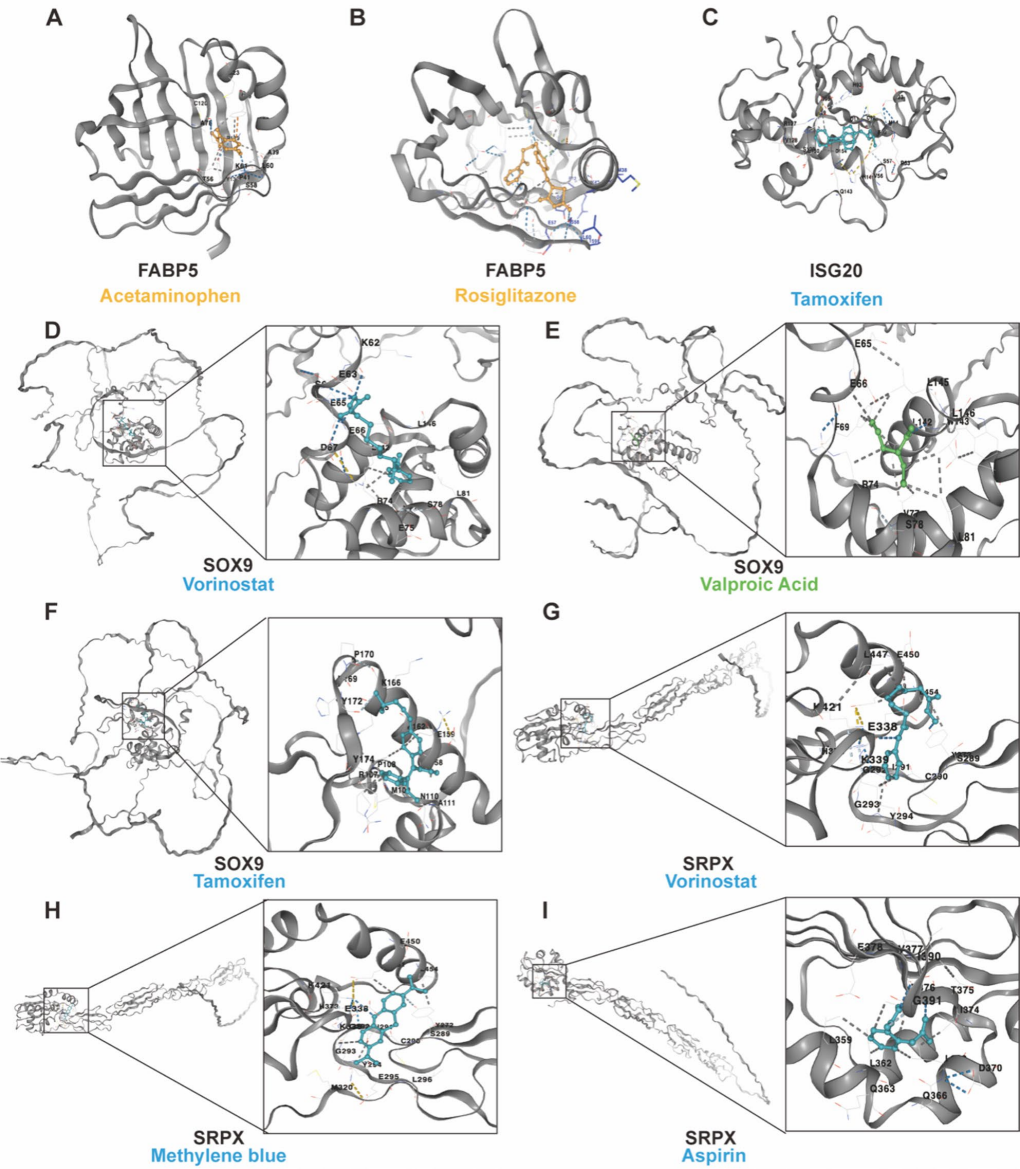
Structural analysis of HGLRGs and potential drugs interactions based on protein–ligand blind docking. Three-dimensional complex structures of FABP5 with acetaminophen (**A**) and rosiglitazone (**B**), and ISG20 with tamoxifen (**C**). Complex structures of SOX9 with vorinostat (**D**), valproic acid (**E**), and tamoxifen (**F**). Complex structures of SRPX with vorinostat (**G**), methylene blue (**H**), and aspirin (**I**)

Given the potential role of HGLRGs in promoting fibrosis progression via immune cell infiltration and extracellular matrix production, we evaluated whether HGLRGs could serve as potential targets for small-molecule drugs in LF treatment. Using the DSigDB database, candidate compounds targeting 7 HGLRGs were identified and screened. Only FDA-approved drugs were selected, prioritizing those with known antifibrotic, antioxidant, or cytoprotective properties, while excluding agents associated with toxicity. Seven candidate drugs were ultimately selected: acetaminophen, rosiglitazone, tamoxifen, vorinostat, valproic acid, methylene blue, and aspirin. Molecular docking simulations using CB-Dock2 predicted the lowest-energy binding conformations between each drug and the active site of the corresponding target protein. All Vina scores were below − 5, indicating strong binding affinity (Supplementary Table 2). Predicted binding interfaces are visualized in Fig. 11, indicating HGLRGs as promising therapeutic targets for LC.

## Discussion

Metabolic reprogramming, particularly hypoxia, glycolysis, and protein lactylation, is increasingly recognized as a driver of liver fibrogenesis. Activated HSCs exhibit enhanced glycolytic flux, lactate accumulation, and dynamic lactylation modifications, which collectively influence energy metabolism, epigenetic regulation, and immune activation [6, 7]. These processes promote extracellular matrix deposition and fibrotic progression. Through integrated transcriptomic analyses, seven key HGLRGs—CHST4, FABP5, GPC3, SOX9, SRPX, IFI16, and ISG20—were identified as consistently upregulated in LF. These genes demonstrated promising diagnostic value, were associated with immune infiltration, and emerged as potential molecular targets for anti-fibrotic drug therapy.

From a mechanistic perspective, HGLRGs appear to contribute to LF through two complementary pathways: direct promotion of matrix deposition and modulation of the hepatic immune microenvironment. Several HGLRGs are established profibrotic regulators. SOX9, a transcription factor activated during chronic liver injury, directly regulates the expression of extracellular matrix genes, including type I collagen, and its expression correlates with fibrosis stage and progression to cirrhosis [15]. In murine models, SOX9 inactivation protects the liver from both parenchymal and cholestatic fibrosis, improving hepatic function and mitigating chronic inflammation [15]. GPC3, co-expressed with α-SMA in fibrotic livers such as biliary atresia, has been proposed as part of a biomarker panel for assessing fibrosis severity [16]. ISG20 is upregulated across multiple fibrotic conditions, including renal fibrosis [17, 18], suggesting involvement in a conserved fibrotic response. Its elevated expression in LC by immunohistochemistry and its ability to promote LX-2 activation and α-SMA expression in vitro further support a profibrotic role. Notably, ISG20 expression appears to vary across fibrosis etiologies, with differences observed in NAFLD-associated fibrosis, suggesting potential distinctions between viral and non-viral fibrogenic pathways. This notion is possibely supported by the observation that ISG20 is inducible by HBV infection and has been closely associated with disease progression and clinical outcomes in HBV-related liver disorders, particularly hepatocellular carcinoma [19]. Future large-scale clinical studies in cirrhosis of varying etiologies—especially non-HBV-related cases—are warranted to validate the diagnostic and prognostic value of ISG20. Emerging evidence suggests that SRPX may participate in tissue and stromal remodeling, as it has been implicated in cardiac remodeling [20] and identified as a prognostic-related gene in cancer-associated fibroblasts in breast cancer [21]. Although its specific functions in LF have yet to be defined, these observations suggest a potential involvement of SRPX in fibrogenic processes and warrant further mechanistic investigation. Combing with functional predictions, HLGRGs are likely to contribute to LF through mechanisms involving extracellular matrix remodeling and pro-fibrotic signaling.

In parallel, several HGLRGs primarily influence fibrogenesis through immune regulation. IFI16, an innate immune DNA sensor, activates TLR4-mediated inflammatory signaling, promotes immune cell recruitment, and drives extracellular matrix remodeling, as demonstrated in skin and cardiac fibrosis [22–25]. CHST4, a carbohydrate sulfotransferase involved in lymphocyte homing [26], has been linked to CD4⁺ T cell and macrophage infiltration in hepatic and extrahepatic diseases [27]. FABP5, a lipid chaperone, is strongly expressed in scar-associated CD9⁺TREM2⁺ macrophages that drive collagen deposition [28, 29]. In liver injury, FABP5⁺ macrophages derived from monocytes promote neutrophil activity and contribute to immune remodeling [30]. Its involvement in fibrosis across multiple organs, including pulmonary arterial hypertension [31] and radiation-induced dermal fibrosis [32] further indicates its broad pathogenic function. Consistent with this, FABP5 was validated to be upregulated in LC by qPCR and immunohistochemistry assays. Collectively, these findings suggest that HGLRGs, particularly FABP5, IFI16, and CHST4, may promote LF via establishing an inflammatory immune microenvironment favorable to fibrogenesis.

From a translational perspective, HGLRGs exhibit promising diagnostic and therapeutic relevance. Several genes, including CHST4, IFI16, SOX9, and SRPX, achieved high discriminatory performance for LF (AUC ≥ 0.8), while FABP5, ISG20, and GPC3 demonstrated moderate diagnostic value. Importantly, combinatorial models incorporating two to three HGLRGs showed improved diagnostic accuracy compared with single-gene markers, with certain combinations exceeding an AUC of 0.86. These findings support the feasibility of multi-gene diagnostic strategies, although validation in independent, large-scale cohorts remains essential. Notably, SRPX, as a secreted protein, represents an attractive candidate for non-invasive disease monitoring. Evaluation of circulating SRPX levels across different chronic LF and fibrosis stages may further enhance the clinical applicability of HGLRG-based biomarkers.

Therapeutically, molecular docking analyses identified several FDA-approved drugs with predicted binding affinity to HGLRGs, providing a rationale for antifibrotic drug repurposing. Histone deacetylase inhibitors, including vorinostat and valproic acid, have been widely shown to suppress HSC activation and reduce extracellular matrix deposition through epigenetic regulation, modulation of the TGF-β/Smad, NF-κB, and EMT pathways [33–38]. Our docking results further demonstrated stable interactions between these agents and SOX9 or SPRX, indicating that HDAC inhibitors may influencing the hypoxia, glycolysis, or lactylation. Nonsteroidal anti-inflammatory drugs also displayed potential antifibrotic relevance. Aspirin has been reported to attenuate LF by inhibiting TGF-β1/Smad signaling [39], reducing IL-1β and COX-2 expression [40], blocking the TLR4/NF-κB pathway [41], and promoting autophagy [42]. Although acetaminophen demonstrated binding potential and antifibrotic activity in cardiac fibrosis [43], its known hepatotoxicity warrants extreme caution. Rosiglitazone also exhibited docking compatibility with HGLRGs, consistent with its known ability to modulate PPAR-γ activity, inhibit NF-κB phosphorylation, and improve fibrosis in BDL-induced mouse models [44]. Experimental data also demonstrate that tamoxifen inactivates HSCs and exerts protective effects in multiple fibrotic models, including DDC-induced LF [45], peritoneal fibrosis [46], and renal fibrosis [47]. Although direct evidence for an antifibrotic effect of methylene blue in the liver is lacking, its beneficial role in postoperative peritoneal adhesions [48] and hepatopulmonary syndrome [49] suggests potential relevance to fibrosis-related processes. Its specific interaction with SRPX identified in this study provides a new lead for future investigation. Collectively, this work not only identifies clinically available compounds that may target HGLRGs but also offers mechanistic support for drug repurposing.

Several limitations should be acknowledged. First, although normalization and batch-effect correction using the ComBat function were applied for batch effects, the residual variability between datasets, including biological and technical heterogeneity, may be unavoidable. Thus, independent validation in larger, well-controlled cohorts remains essential. Second, only seven HGLRGs were identified, which may be related to the characteristics of the datasets used. For instance, the GSE84044 dataset incorporated samples from HBV-infected individuals rather than completely healthy controls, potentially excluding genes involved in the shared inflammatory pathways of HBV-related fibrosis and cirrhosis. Moreover, due to the limited number of candidate genes and the lack of prognostic information in available data, it was not feasible to develop a comprehensive LASSO‑based risk score model. Third, the actual binding properties of the candidate drugs (screened via molecular docking) to target proteins, their inhibitory effects on HSC activation, and their in vivo anti‑fibrotic efficacy require further experimental and pharmacological validation. Fourth, only FABP5 and ISG20 expression were preliminarily validated in LX‑2 cells and human cirrhotic tissues. The specific molecular functions and upstream or downstream regulatory networks of the remaining HGLRGs in LF await further in vitro and in vivo verification. Fifth, most datasets included were derived from viral hepatitis-associated fibrosis. Although partial validation was performed in a NAFLD cohort, more validation across etiologically diverse Lf cohorts is needed to generalize the conclusions. Sixth, although the CIBERSORT algorithm suggested that HGLRGs influence immune infiltration in LF, dynamic immune cell infiltration patterns varied across cohorts, likely due to differences in etiology, disease stage, and sample processing. Despite consistent observations—such as the negative correlations of ISG20 with resting NK cells and of SOX9 with M2 macrophages—further validation in larger, stable cohorts is warranted. Lastly, SRPX is a secreted protein and represents an ideal non‑invasive biomarker. Assessing circulating SRPX levels across various chronic liver diseases and fibrosis stages may enhance the translational diagnostic utility of HGLRGs in LF.

## Conclusion

Collectively, seven HGLRGs associated with the diagnosis and progression of LF were identified. These genes may promote hepatic fibrosis by modulating liver metabolism, extracellular matrix production, and immune cell infiltration within the tumor microenvironment. Furthermore, seven candidate drugs, including rosiglitazone, were screened as potential therapeutic agents for LF. This study provides a novel theoretical foundation for the risk stratification and precision treatment of LC.

## Electronic supplementary material

Below is the link to the electronic supplementary material.

Supplementary Material 1

## Data Availability

The datasets analyzed in this study are publicly available data and annotated with the source. The data and material used during the current study are available from the corresponding author upon reasonable request.

## Abbreviations

AUC: Area under the curve
CHST4: Carbohydrate sulfotransferase 4
DEGs: Differentially expressed genes
GEO: Gene Expression Omnibus
GO: Gene Ontology
GPC3: Glypican-3
GSEA: Gene Set Enrichment Analysis
HE: Hematoxylin-eosin
FABP5: Fatty acid-binding protein 5
HGLRGs: Hypoxia: glycolysis and lactylation-related genes
HSCs: Hepatic stellate cells
IFI16: Interferon gamma inducible protein 16
IHC: Immunohistochemistry
ISG20: Interferon-stimulated exonuclease gene 20
LC: Liver cirrhosis
LF: Liver fibrosis
KEGG: Kyoto Encyclopedia of Genes and Genomes
NAFLD: Non-alcoholic fatty liver disease
ROC: Receiver operating characteristic
SOX9: SRY-related HMG-box transcription factor 9
SRPX: Sushi repeat-containing protein X-linked
WB: Western blotting
WGCNA: Weighted gene co-expression network analysis
