# Supplementary material for: The impact of hypoxia and glycolysis on liver fibrosis
Source: J Transl Med. 2026 May 16;24:875. doi: 10.1186/s12967-026-08147-5 (PMC13352929; doi:10.1186/s12967-026-08147-5)
Supplement: Supplementary file 1 — Supplementary Material 1 [file 12967_2026_8147_MOESM1_ESM.docx]

**Supplementary figures:**


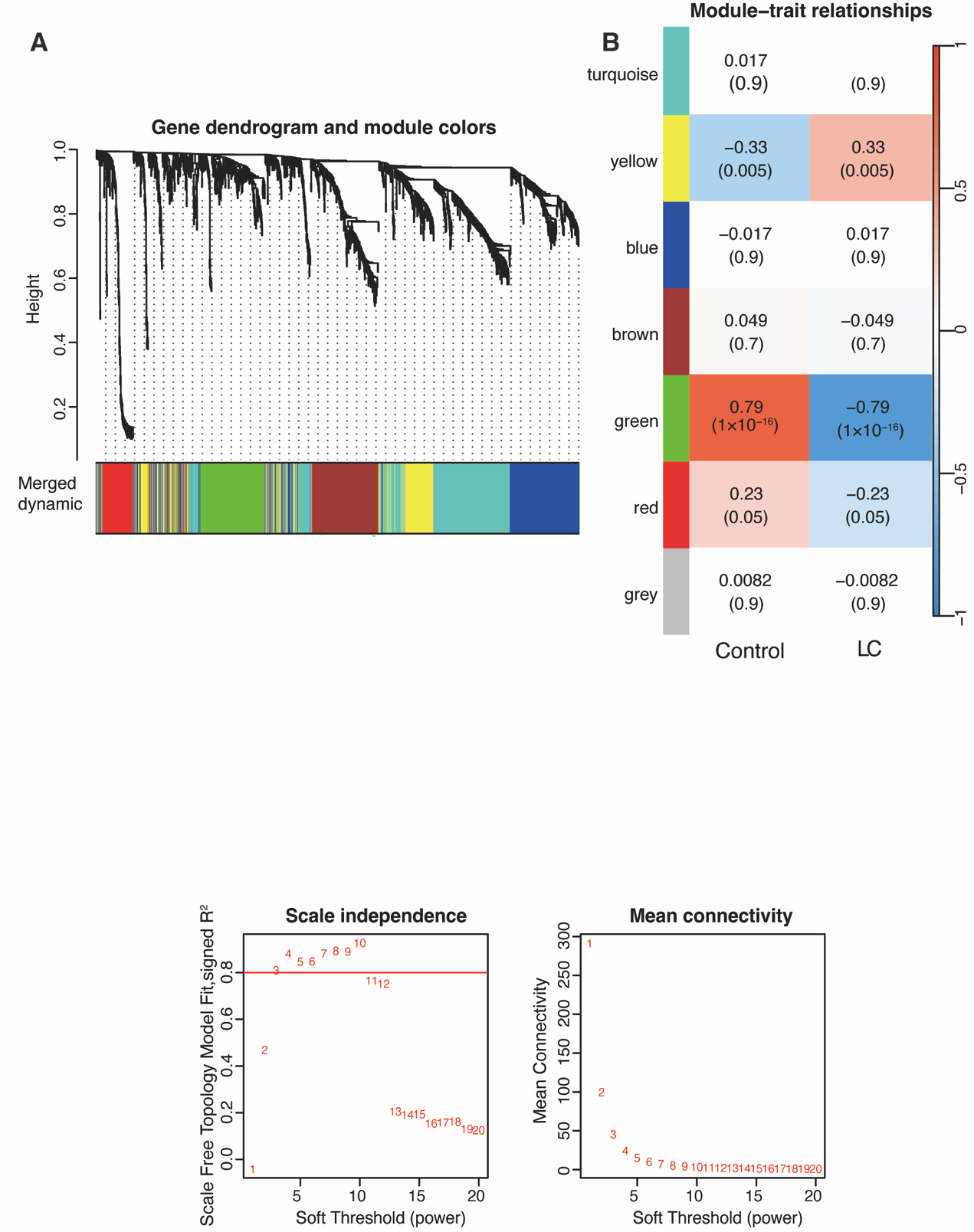


Supplementary figure 1: WGCNA reveals modules associated with LF.

(A) Gene dendrogram showing hierarchical clustering of genes based on topological overlap, with different modules assigned unique colors. (B) Heatmap of module-trait relationships. The correlation coefficients and corresponding *p*-values between each module and clinical traits are shown.


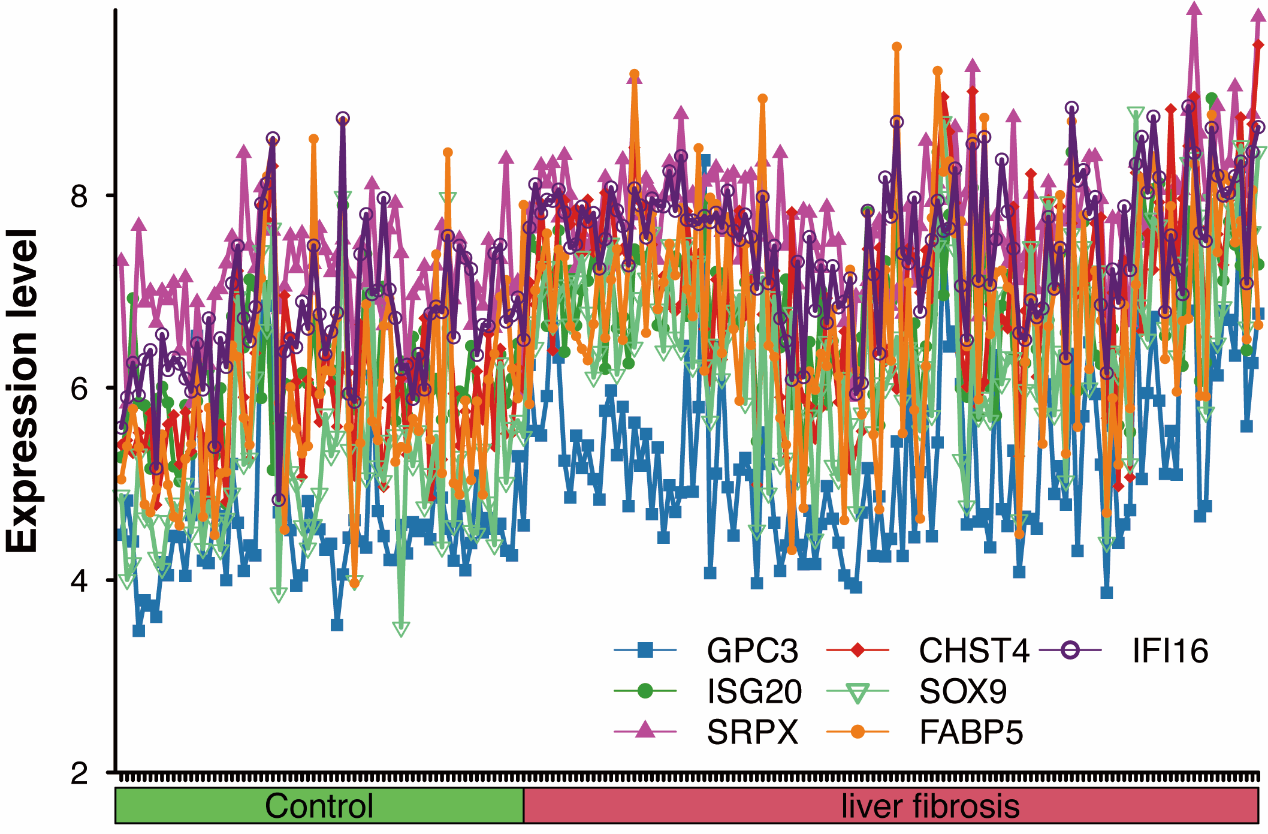


Supplementary figure 2: Expression profiles of HGLRGs in LF and control samples.

Line plot showing the expression levels of seven selected HGLRGs—CHST4, IFI16, SRPX, SOX9, FABP5, ISG20, and GPC3—across samples in control (green) and liver fibrosis (red) groups. Each gene is represented by a distinct color and symbol.


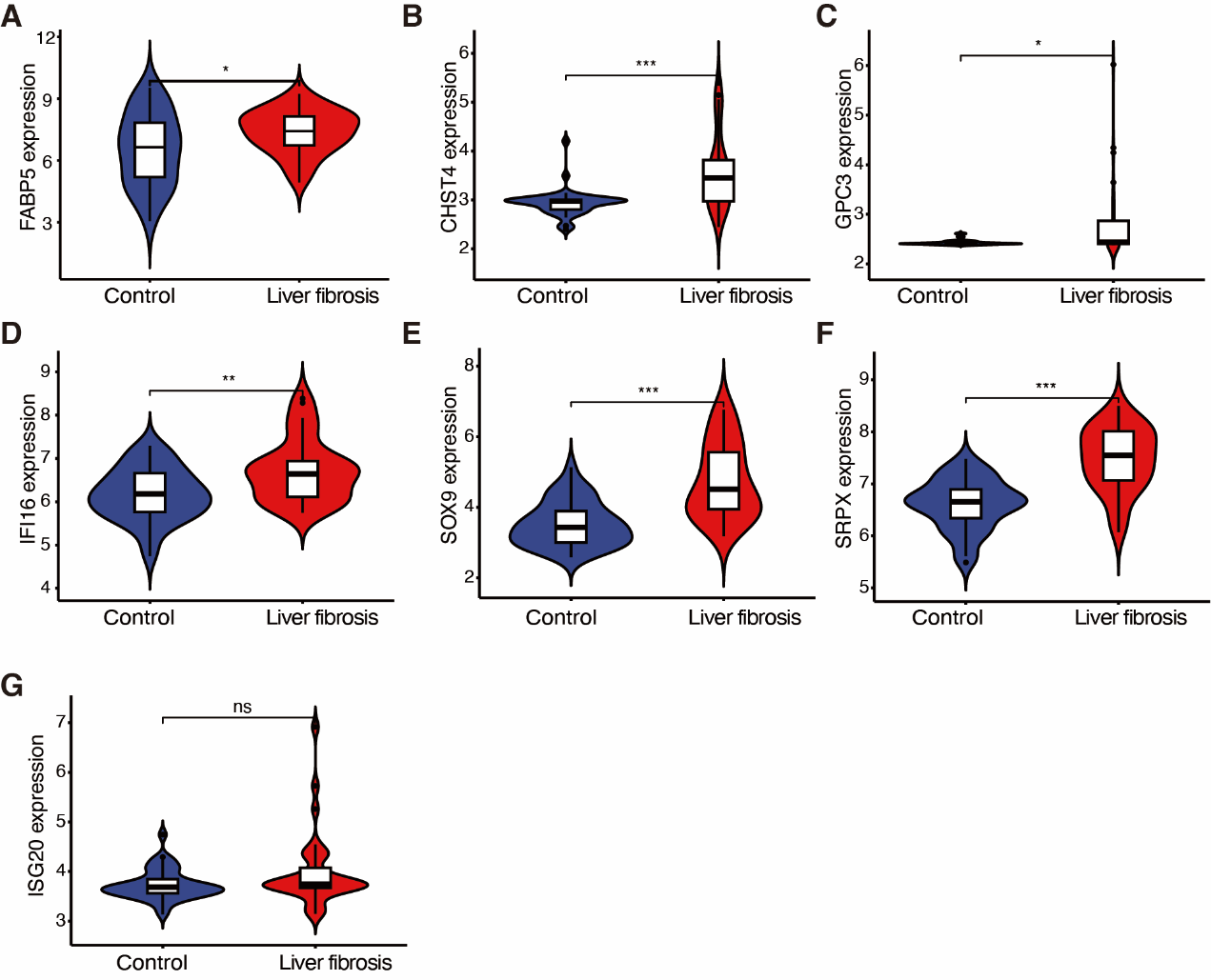


Supplementary figure 3: Violin plots showing the expression levels of differentially expressed HGLRGs in GSE49541.


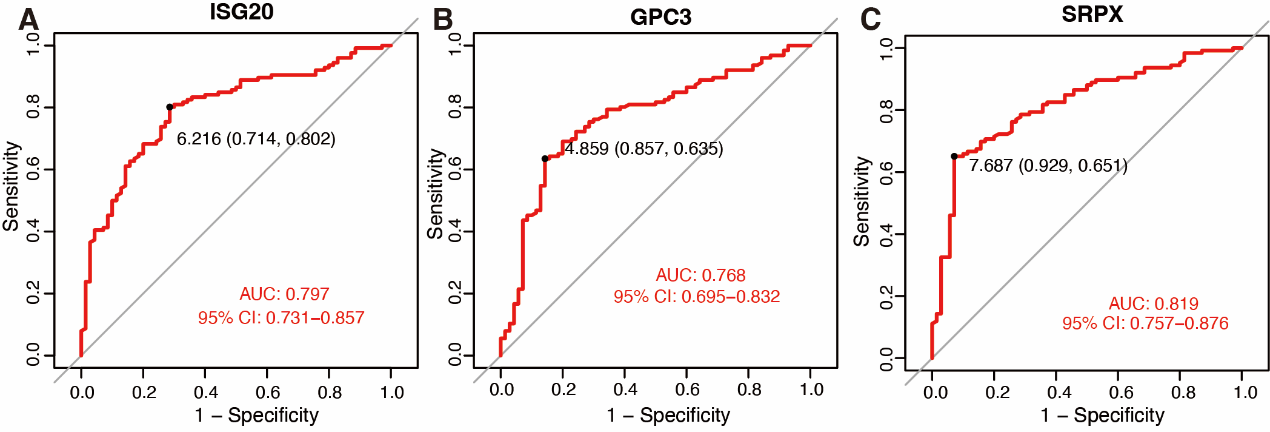


Supplementary figure 4: ROC curve analyses of ISG20 (A), GPC3 (B) and SRPX (C) in LF.


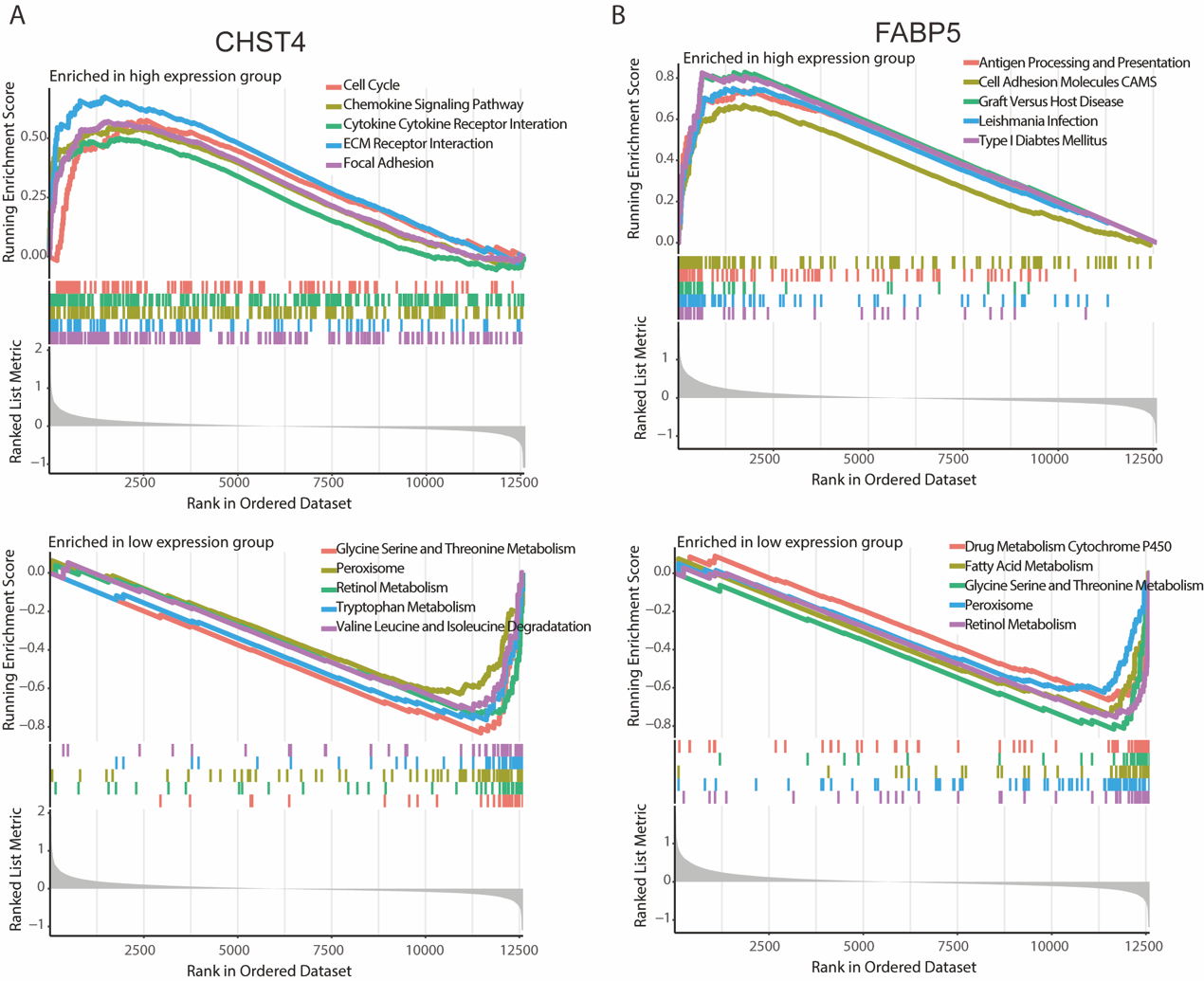


Supplementary figure 5: GSEA of CHST4 and FABP5 expression in LF


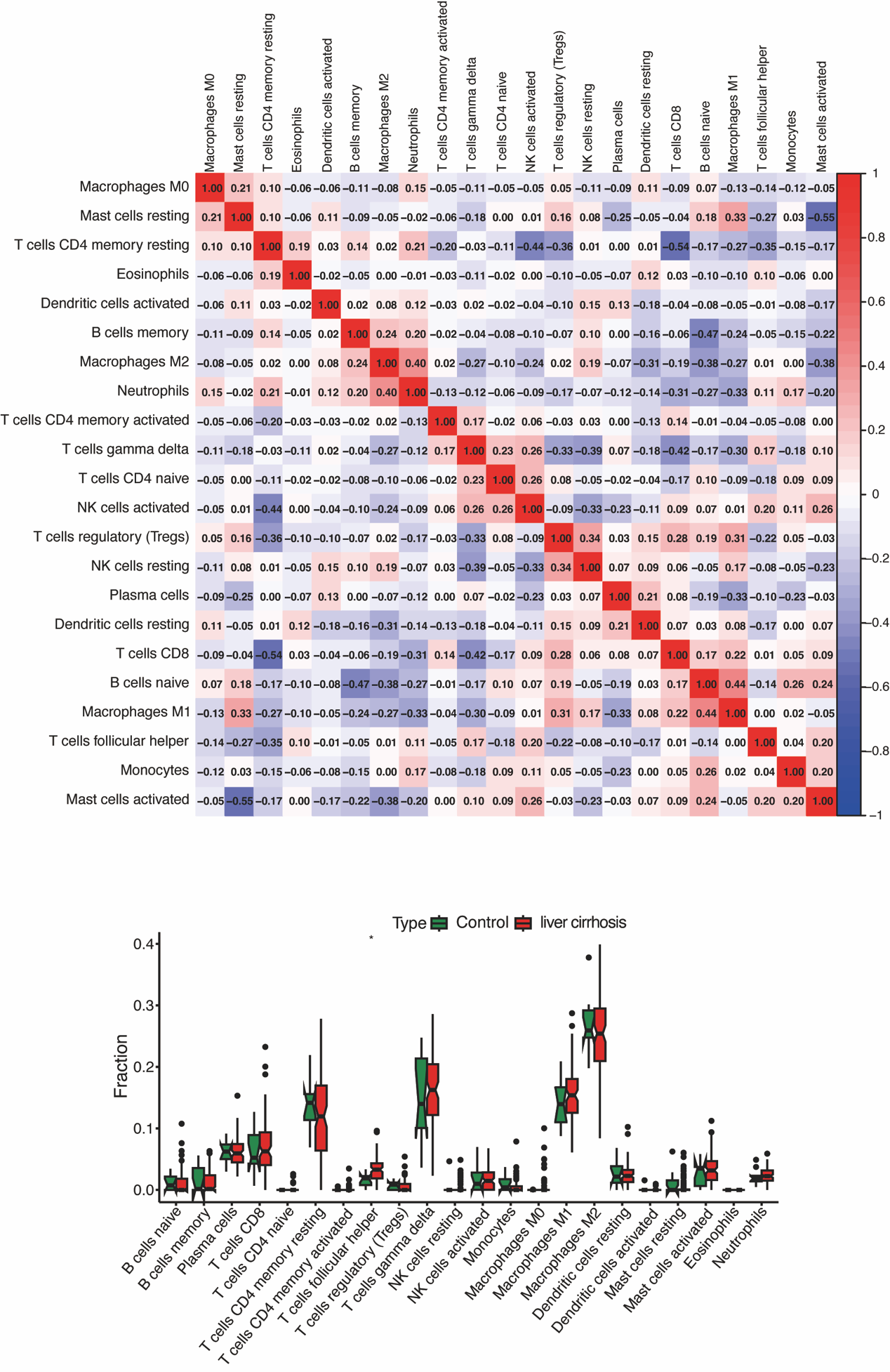


Supplementary figure 6: Immune cell infiltration landscape in LF samples.

Correlation heatmap of the 22 immune cell types. The values represent Pearson correlation coefficients, with red indicating positive correlations and blue indicating negative correlations.


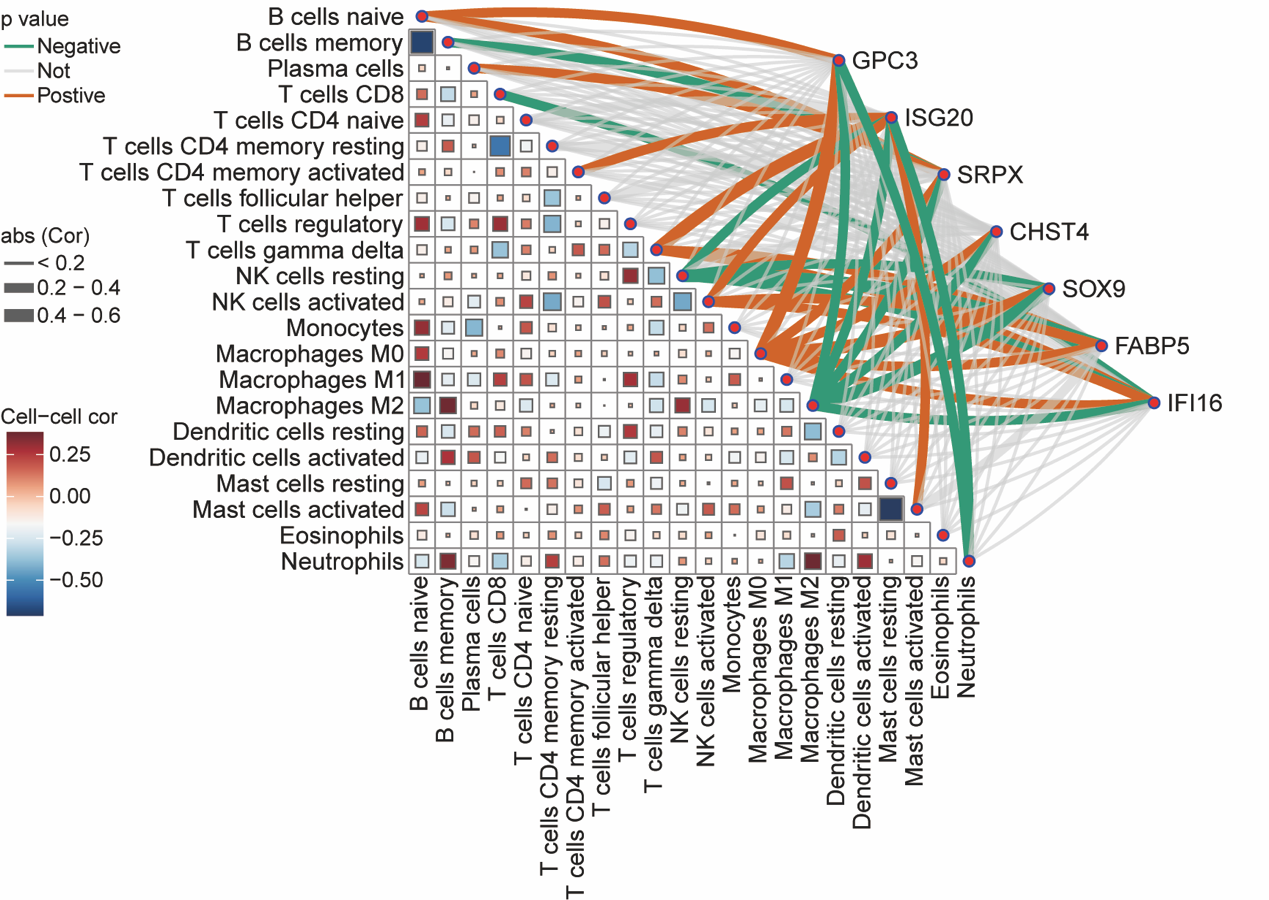


Supplementary figure 7: Correlation analysis between HGLRGs and immune cell infiltration in LF.

A network heatmap showing the relationships between seven HGLRGs (GPC3, ISG20, SRPX, CHST4, SOX9, FABP5, and IFI16) and 22 immune cell types based on CIBERSORTx deconvolution analysis. The square matrix in the center represents the cell–cell correlation (cell-cell cor), where color intensity indicates the correlation coefficient (red for positive, blue for negative) and the thickness of gray edges reflects the absolute correlation (abs(Cor)). The outer arcs visualize the gene–immune cell correlations: orange edges represent significant positive correlations, green edges represent significant negative correlations, and gray lines indicate non-significant associations.


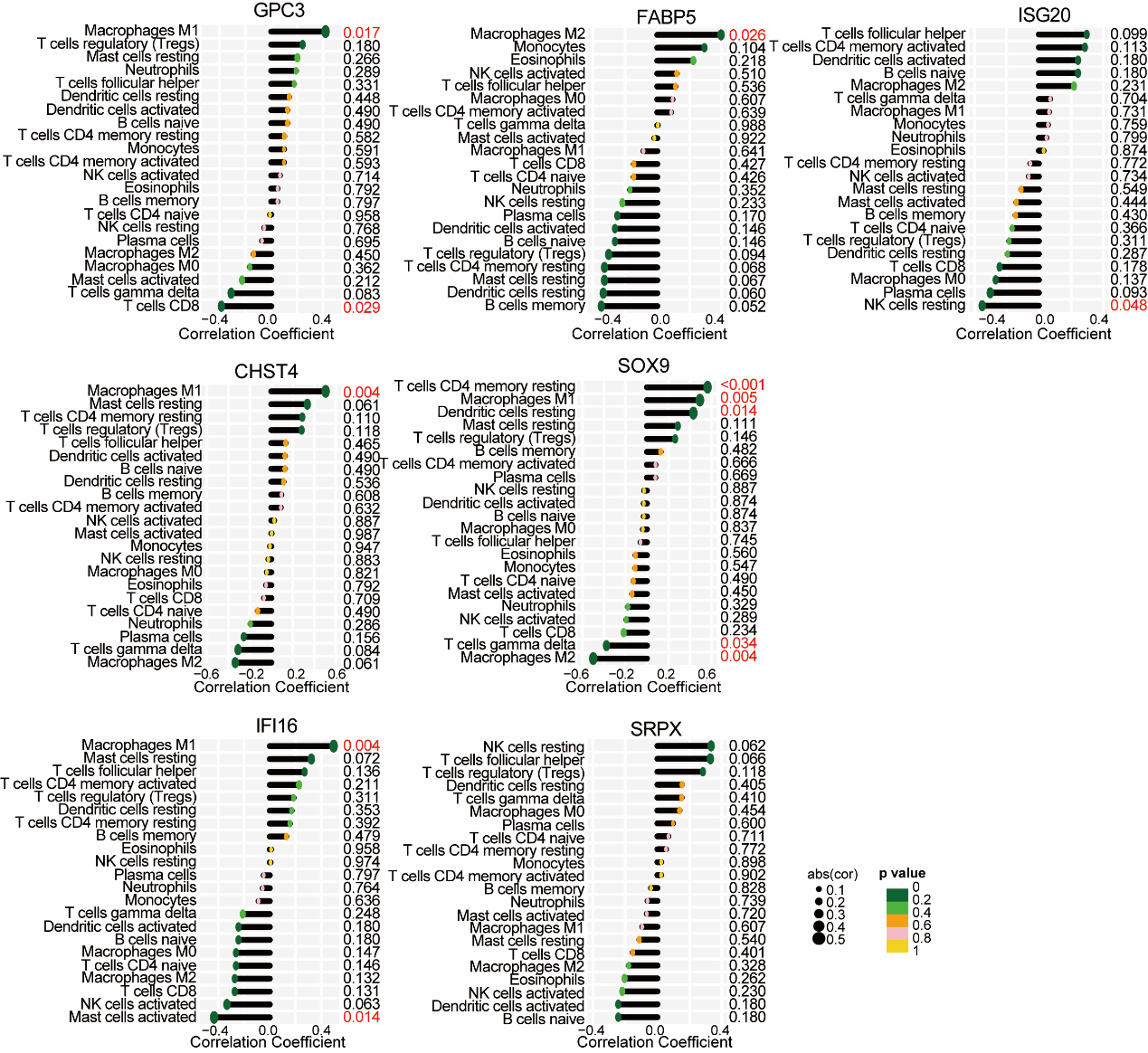


Supplementary figure 8: Spearman correlation analysis between 7 HGLRGs expression and immune cell infiltration in GSE49541 by CIBERSORT.

Supplementary Table 1: Diagnostic performance of single and combined HGLRGs for LF

| **Genes** | **Numbers of genes** | **AUC** | **Threshold** | **Sensitivity** | **Specificity** |
| --- | --- | --- | --- | --- | --- |
| SOX9 | 1 | 0.853 | 5.69 | 0.833 | 0.843 |
| CHST4 | 1 | 0.849 | 6.57 | 0.738 | 0.886 |
| IFI16 | 1 | 0.829 | 7.025 | 0.81 | 0.771 |
| SRPX | 1 | 0.819 | 7.687 | 0.651 | 0.929 |
| ISG20 | 1 | 0.797 | 6.216 | 0.802 | 0.714 |
| GPC3 | 1 | 0.768 | 4.859 | 0.635 | 0.857 |
| FABP5 | 1 | 0.766 | 6.212 | 0.746 | 0.729 |
| SRPX SOX9 | 2 | 0.868 | 6.72 | 0.762 | 0.871 |
| ISG20 CHST4 | 2 | 0.866 | 6.731 | 0.698 | 0.914 |
| ISG20 SOX9 | 2 | 0.866 | 6.117 | 0.786 | 0.843 |
| SRPX CHST4 | 2 | 0.866 | 7.076 | 0.738 | 0.914 |
| CHST4 IFI16 | 2 | 0.866 | 6.678 | 0.849 | 0.843 |
| CHST4 SOX9 | 2 | 0.863 | 5.759 | 0.857 | 0.8 |
| SOX9 IFI16 | 2 | 0.862 | 6.172 | 0.881 | 0.786 |
| SRPX IFI16 | 2 | 0.86 | 7.385 | 0.738 | 0.829 |
| GPC3 SOX9 | 2 | 0.849 | 5.057 | 0.849 | 0.8 |
| ISG20 IFI16 | 2 | 0.84 | 7.021 | 0.635 | 0.914 |
| SOX9 FABP5 | 2 | 0.84 | 5.984 | 0.802 | 0.786 |
| GPC3 IFI16 | 2 | 0.839 | 6.13 | 0.683 | 0.886 |
| GPC3 CHST4 | 2 | 0.838 | 5.635 | 0.762 | 0.843 |
| CHST4 FABP5 | 2 | 0.836 | 6.497 | 0.722 | 0.857 |
| GPC3 SRPX | 2 | 0.829 | 6.173 | 0.746 | 0.843 |
| GPC3 ISG20 | 2 | 0.816 | 5.767 | 0.675 | 0.886 |
| SRPX FABP5 | 2 | 0.815 | 6.813 | 0.794 | 0.757 |
| FABP5 IFI16 | 2 | 0.806 | 6.432 | 0.865 | 0.714 |
| ISG20 SRPX CHST4 | 3 | 0.88 | 6.861 | 0.762 | 0.871 |
| ISG20 SRPX SOX9 | 3 | 0.88 | 6.655 | 0.77 | 0.871 |
| SRPX CHST4 IFI16 | 3 | 0.877 | 7.103 | 0.77 | 0.9 |
| SRPX CHST4 SOX9 | 3 | 0.873 | 6.667 | 0.746 | 0.9 |
| SRPX SOX9 IFI16 | 3 | 0.872 | 6.771 | 0.794 | 0.857 |
| ISG20 CHST4 SOX9 | 3 | 0.87 | 6.34 | 0.762 | 0.886 |
| ISG20 CHST4 IFI16 | 3 | 0.868 | 6.466 | 0.865 | 0.786 |
| ISG20 SOX9 IFI16 | 3 | 0.867 | 6.155 | 0.881 | 0.729 |
| GPC3 CHST4 IFI16 | 3 | 0.865 | 6.189 | 0.786 | 0.871 |
| ISG20 SRPX IFI16 | 3 | 0.865 | 7.16 | 0.698 | 0.9 |
| CHST4 SOX9 IFI16 | 3 | 0.865 | 6.2 | 0.865 | 0.814 |
| GPC3 SRPX SOX9 | 3 | 0.863 | 6.024 | 0.77 | 0.871 |
| GPC3 SRPX IFI16 | 3 | 0.859 | 6.471 | 0.77 | 0.871 |
| GPC3 CHST4 SOX9 | 3 | 0.858 | 5.362 | 0.865 | 0.771 |
| GPC3 SOX9 IFI16 | 3 | 0.858 | 5.566 | 0.905 | 0.729 |
| GPC3 ISG20 CHST4 | 3 | 0.857 | 6.097 | 0.714 | 0.929 |
| GPC3 ISG20 SOX9 | 3 | 0.857 | 5.81 | 0.73 | 0.9 |
| SRPX SOX9 FABP5 | 3 | 0.855 | 6.347 | 0.833 | 0.757 |
| CHST4 SOX9 FABP5 | 3 | 0.854 | 6.244 | 0.778 | 0.857 |
| GPC3 SRPX CHST4 | 3 | 0.852 | 6.421 | 0.698 | 0.943 |
| SRPX CHST4 FABP5 | 3 | 0.852 | 6.789 | 0.754 | 0.857 |
| ISG20 SOX9 FABP5 | 3 | 0.847 | 6.118 | 0.794 | 0.786 |
| CHST4 FABP5 IFI16 | 3 | 0.847 | 6.609 | 0.778 | 0.843 |
| SOX9 FABP5 IFI16 | 3 | 0.846 | 6.344 | 0.81 | 0.786 |
| GPC3 SOX9 FABP5 | 3 | 0.845 | 5.518 | 0.81 | 0.786 |
| ISG20 CHST4 FABP5 | 3 | 0.844 | 6.383 | 0.778 | 0.8 |
| GPC3 CHST4 FABP5 | 3 | 0.843 | 6.039 | 0.722 | 0.871 |
| SRPX FABP5 IFI16 | 3 | 0.837 | 6.92 | 0.794 | 0.786 |
| ISG20 SRPX FABP5 | 3 | 0.833 | 6.694 | 0.77 | 0.786 |
| GPC3 SRPX FABP5 | 3 | 0.828 | 6.096 | 0.778 | 0.8 |
| GPC3 FABP5 IFI16 | 3 | 0.824 | 5.799 | 0.865 | 0.714 |
| ISG20 FABP5 IFI16 | 3 | 0.823 | 6.531 | 0.81 | 0.771 |
| GPC3 ISG20 FABP5 | 3 | 0.817 | 5.978 | 0.698 | 0.829 |

Supplementary Table 2: Molecular docking analysis of candidate drugs with their respective targets.

| **Drugs** | **Primary clinical indication** | **Hepatic safety** | **Preclinical antifibrotic efficacy** | **Targets** | **Vina score** | **Cavity volume** | **Contact residues** |
| --- | --- | --- | --- | --- | --- | --- | --- |
| Aspirin | Antiplatelet, antipyretic-analgesic, antirheumatic | Low but existent risk | Inhibition of liver fibrosis progression [38] | SRPX | -5.5 | 330 | Gly292, Gly293, Met320, Asn321, Val322, Phe336, Arg340, Leu359, Gly360, Leu362, Gln363, Gln366, Leu369, Asp370, Ile374, Thr375, Val376, Val377, Glu378, Arg389, Ile390, Gly391, Ala392, Lys393 |
| Methylene blue | Antidote, sepsis, vasodilatory shock, diagnostic agent | Low risk | Reduction of intra-abdominal adhesions [47]^,^ improvement in hepatopulmonary syndrome [48] | SRPX | -6.5 | 359 | Tyr272, Ser289, Cys290, Ile291, Gly292, Gly293, Tyr294, Glu295, Leu296, Gly298, Pro300, Met320, Glu338, Lys339, Arg340, His373, Lys421, Leu447, Glu450, Glu451, Leu454 |
| Tamoxifen | Endocrine therapy for breast cancer | Moderate risk; monitoring required | Anti-fibrotic effects in liver [44]^,^ reduction of renal [46] and peritonea [45] fibrosis | SOX9 | -6.4 | 149 | Arg107, Pro108, Met109, Asn110, Ala111, Ala158, Glu159, Arg162, Val163, His165, Lys166, His169, Pro170, Asp171, Tyr172, Lys173, Tyr174, Phe270, Arg271 |
|  |  |  |  | ISG20 | -5.9 | 338 | Asp11, Cys12, Glu13, Met14, Arg21, Ser23, Asp51, Arg53, Val56, Ser57, His89, Asp90, His93, Asp94, Arg127, Val128, Ser129, Leu130, Arg131, Gln143, His149, Asp154 |
| Vorinostat | Cutaneous T-cell lymphoma | Clinically used, manageable hepatotoxicity | Amelioration of fibrosis in multiple organs(e.g., liver [35], cornea [49], lung [50]) | SRPX | -6 | 359 | Tyr272, Phe288, Ser289, Cys290, Ile291, Gly292, Gly293, Tyr294, Glu295, Leu296, Met320, Glu338, Lys339, Arg340, His373, Lys421, Leu447, Glu450, Glu451, Leu454 |
|  |  |  |  | SOX9 | -5.8 | 184 | Lys62, Glu63, Ser64, Glu65, Glu66, Asp67, Val71, Ile73, Arg74, Glu75, Val77, Ser78, Leu81, Lys82, Gly83, Leu142, Leu145, Leu146, Asn147, Ser149, Glu150, Pro153, Phe154 |
| Valproic Acid | Antiepileptic, mood stabilizer (bipolar disorder), migraine prophylaxis | High risk; routine monitoring required | Attenuation of fibrosis in multiple organs (e.g., liver [51], kidney, lung [52]) | SOX9 | -4.2 | 184 | Glu65, Glu66, Phe69, Ile73, Arg74, Glu75, Val77, Ser78, Leu81, Trp115, Leu142, Trp143, Leu145, Leu146, Asn147, Glu150 |
| Rosiglitazone | Antihyperglycemic | Significant risk; use strictly restricted | Alleviation of BDL-induced liver fibrosis in mice [43] | FABP5 | -7.8 | - | Phe19, Tyr22, Met23, Leu26, Val28, Leu32, Met35, Gly36, Ala39, Pro41, Cys43, Ile54, Thr56, Ser58, Leu60, Lys61, Thr62, Thr63, Phe65, Glu75, Thr77, Ala78, Asp79, Arg81, Gln96, Gln98, Ile107, Arg109, Val118, Cys120, Arg129, Tyr131 |
| Acetaminophen | Antipyretic, analgesi | Wide therapeutic window; high overdose toxicit | Alleviationn of doxorubicin-induced cardiac fibrosis [42] | FABP5 | -5.1 | 104 | Arg10, Arg12, Phe19, Met23, Leu32, Met35, Gly36, Met38, Ala39, Lys40, Pro41, Asp42, Thr56, Glu57, Ser58, Thr59, Leu60, Lys61, Thr62, Thr63, Ala78, Asp79, Cys120, Arg129, Tyr131 |
